# Supplementary material for: Synthesis and Biological Evaluation of Imidazo[2,1-b]Thiazole based Sulfonyl Piperazines as Novel Carbonic Anhydrase II Inhibitors
Source: Metabolites. 2020 Mar 31;10(4):136. doi: 10.3390/metabo10040136 (PMC7240968; doi:10.3390/metabo10040136)

Synthesis and biological evaluation of imidazo[2,1-*b*]thiazole based sulfonyl piperazines as novel carbonic anhydrase II inhibitors

Kesari Lakshmi Manasa[1], Sravya Pujitha[1] , Aaftaab Sethi[1], Arifuddin Mohammed[1, 2,] Mallika Alvala[1*], Andrea Angeli[3], Claudiu T. Supuran[3*]

[1]Department of Medicinal Chemistry, National Institute of Pharmaceutical Education and Research (NIPER), Hyderabad-500 037, India; mallikaalvala@yahoo.in

[2]Department of Chemistry, Anwarul Uloom College, 11-3-918, New Malleypally, Hyderabad-500001, T. S., India (Present Address)

[3]Università degli Studi di Firenze, Neurofarba Dept., Sezione di Scienze Farmaceutiche e Nutraceutiche, Via Ugo Schiff 6, 50019 Sesto Fiorentino, Florence, Italy

Content **Page No**

1. Copies of NMR spectras 2-25

Copy of ^{1}H NMR and ^{13}C NMR spectra of **9aa**

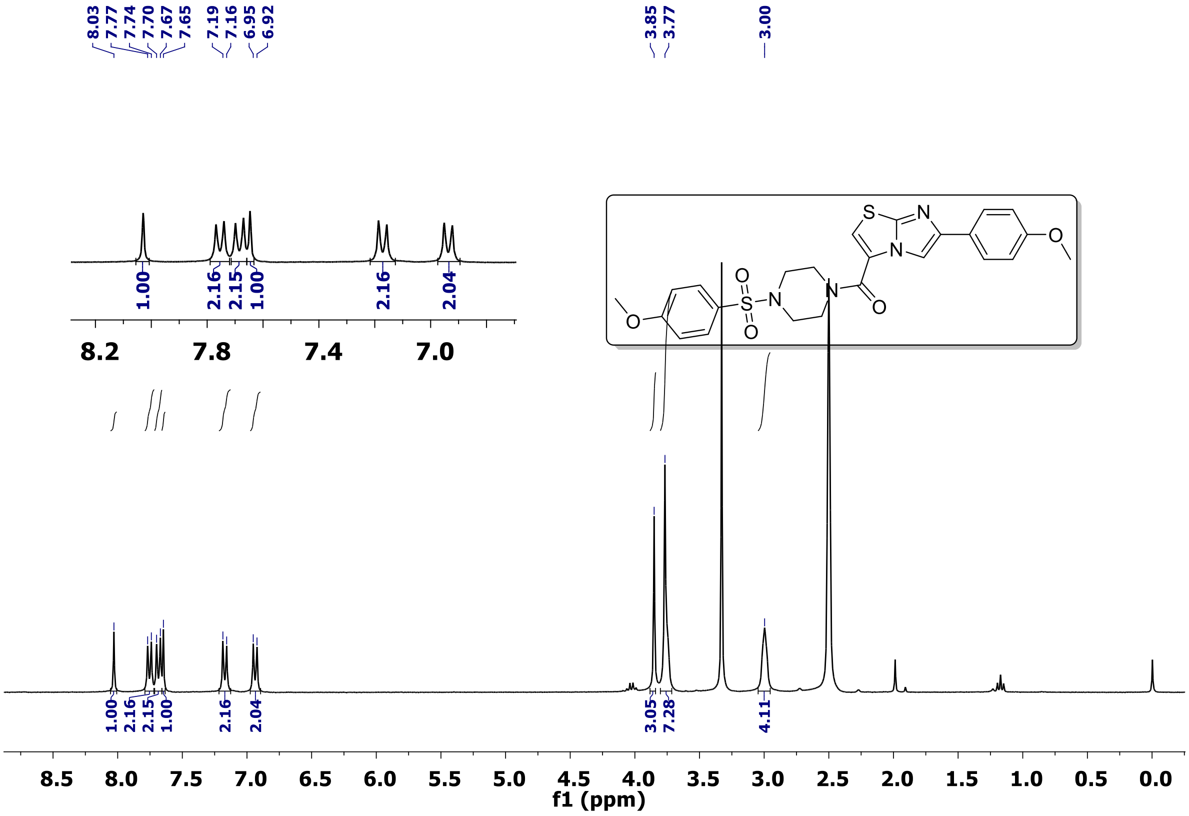

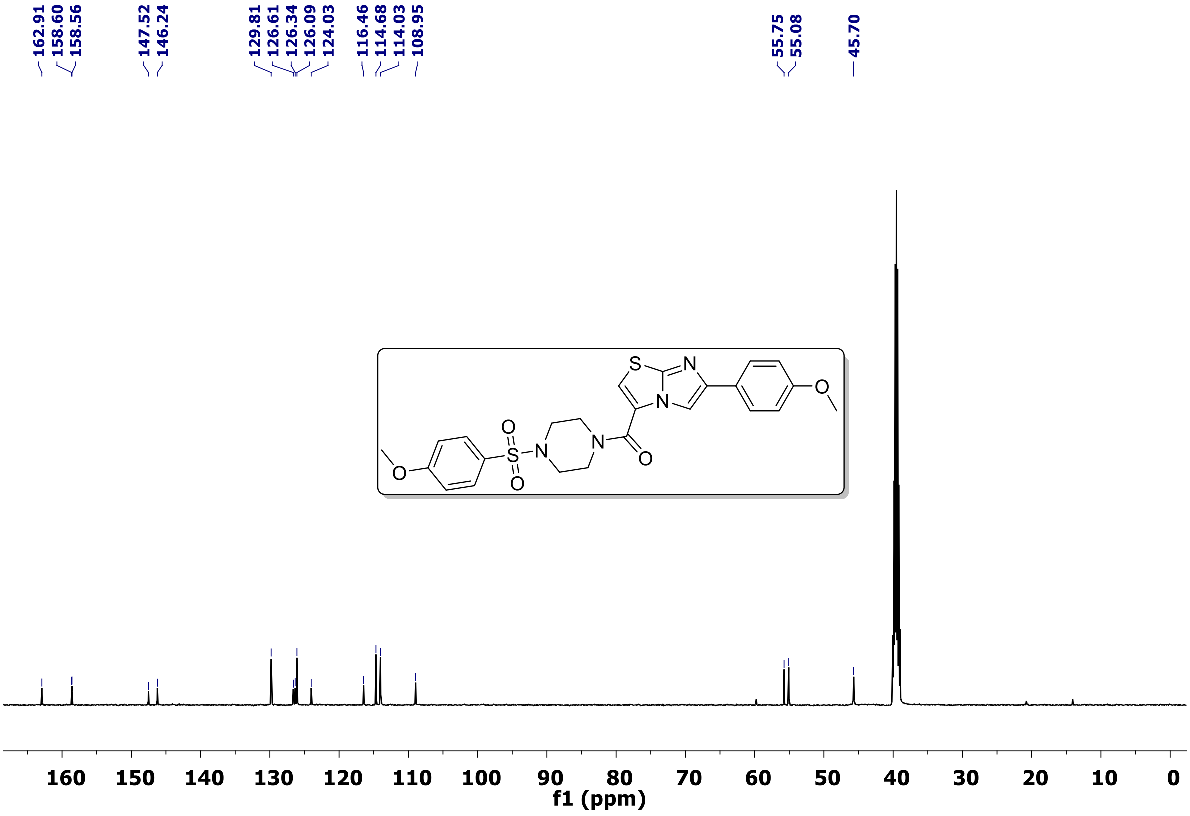

Copy of ^{1}H NMR and ^{13}C NMR spectra of **9ab**

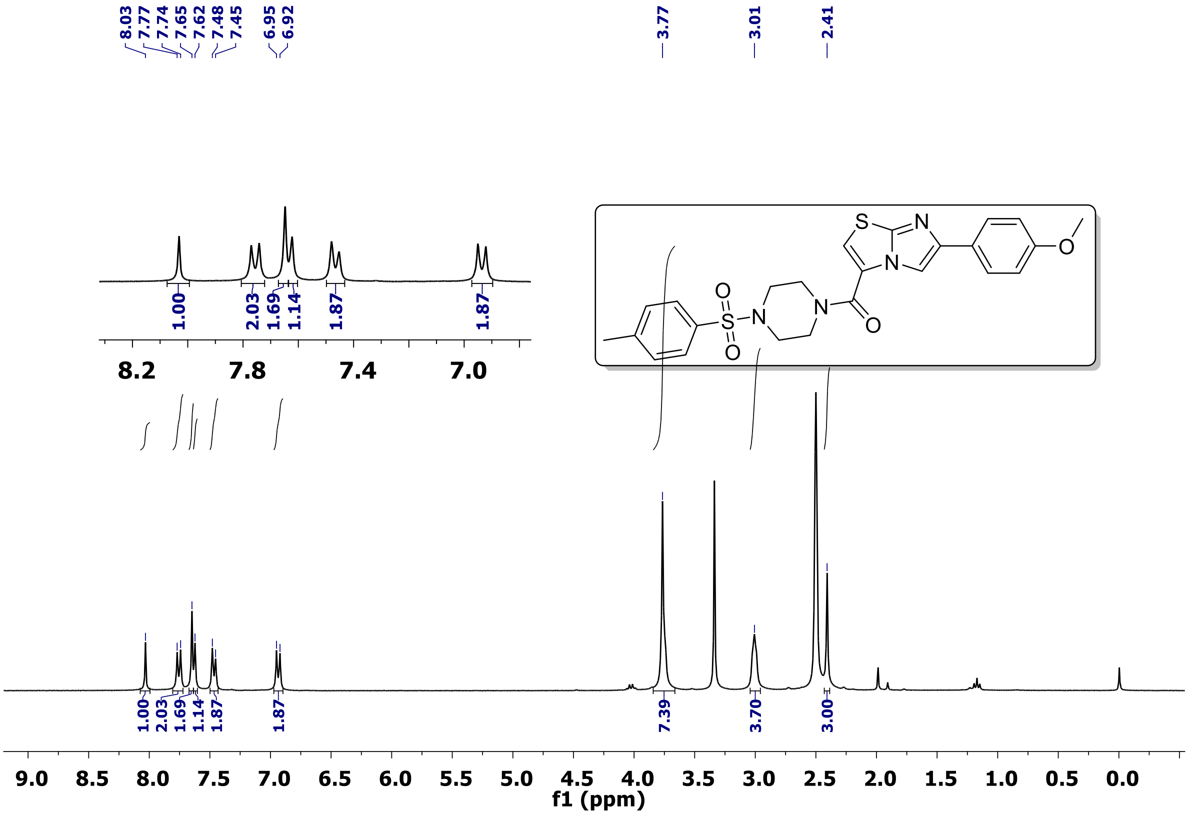

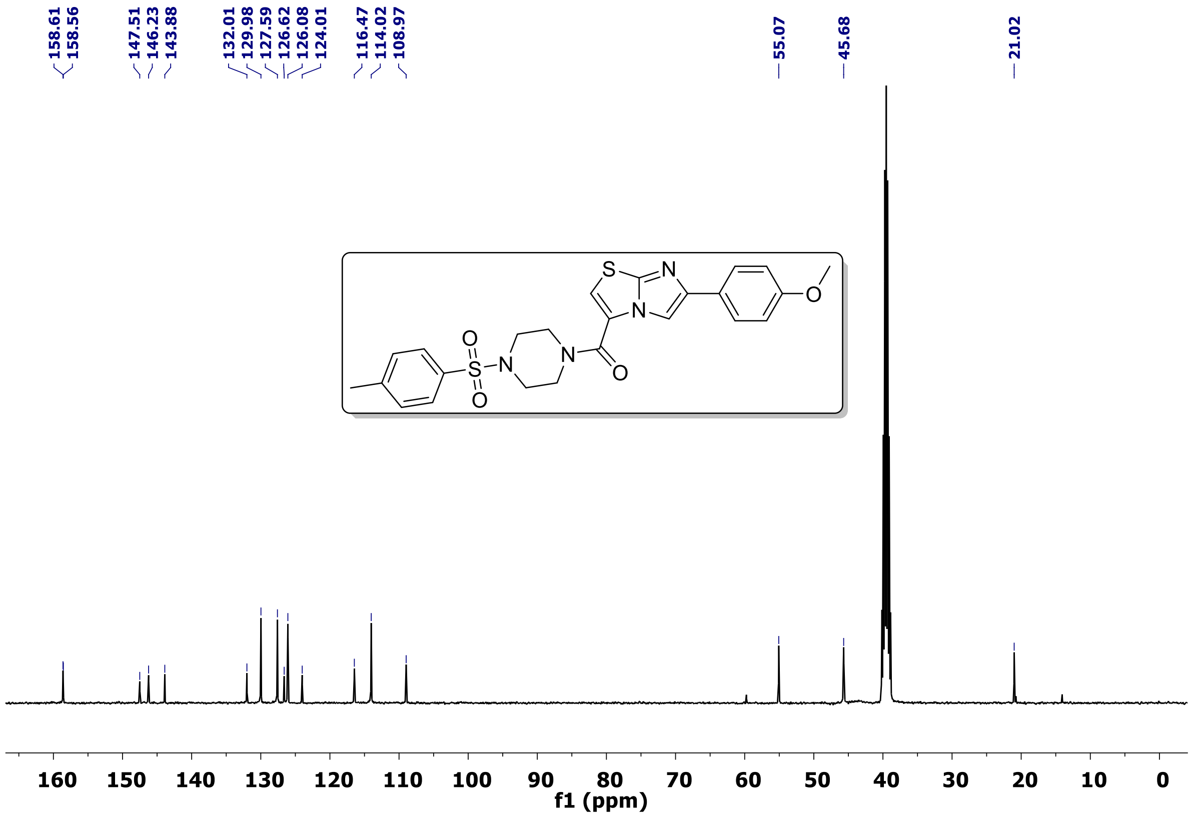

Copy of ^{1}H NMR and ^{13}C NMR spectra of **9ac**

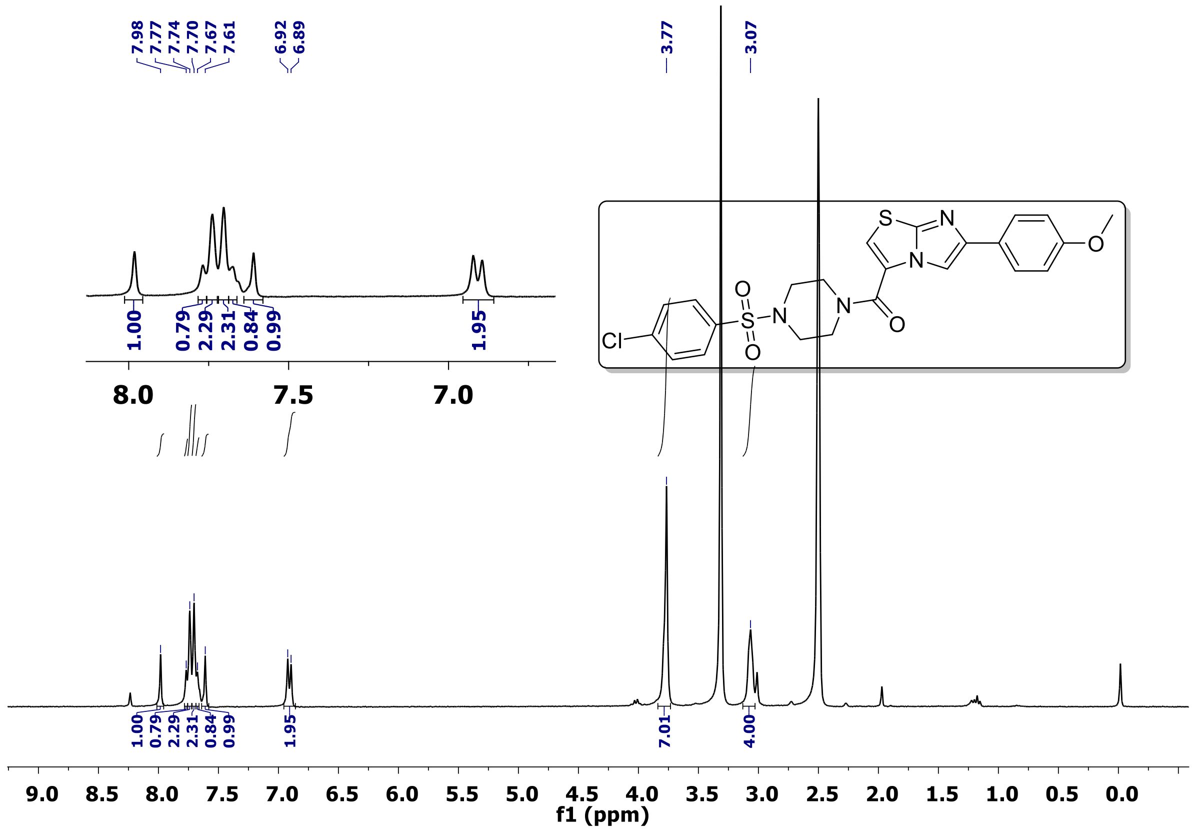

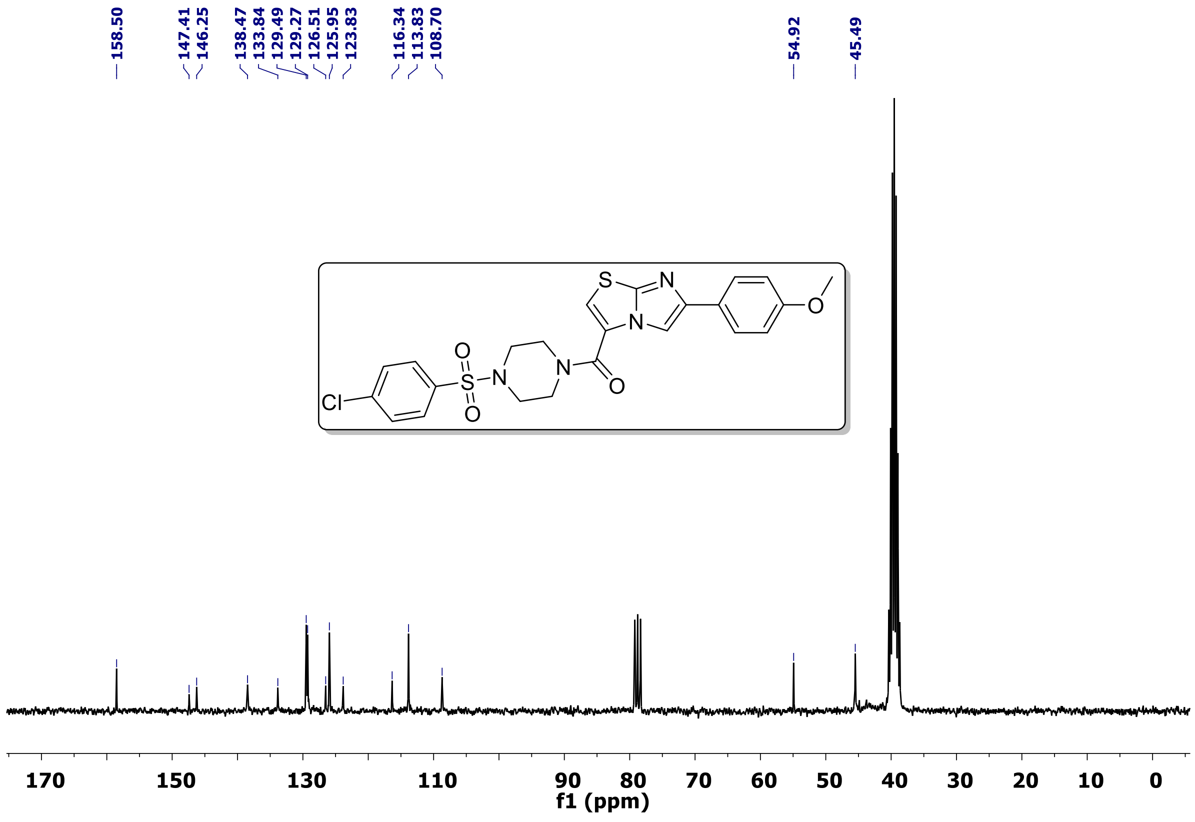

Copy of ^{1}H NMR and ^{13}C NMR spectra of **9ad**

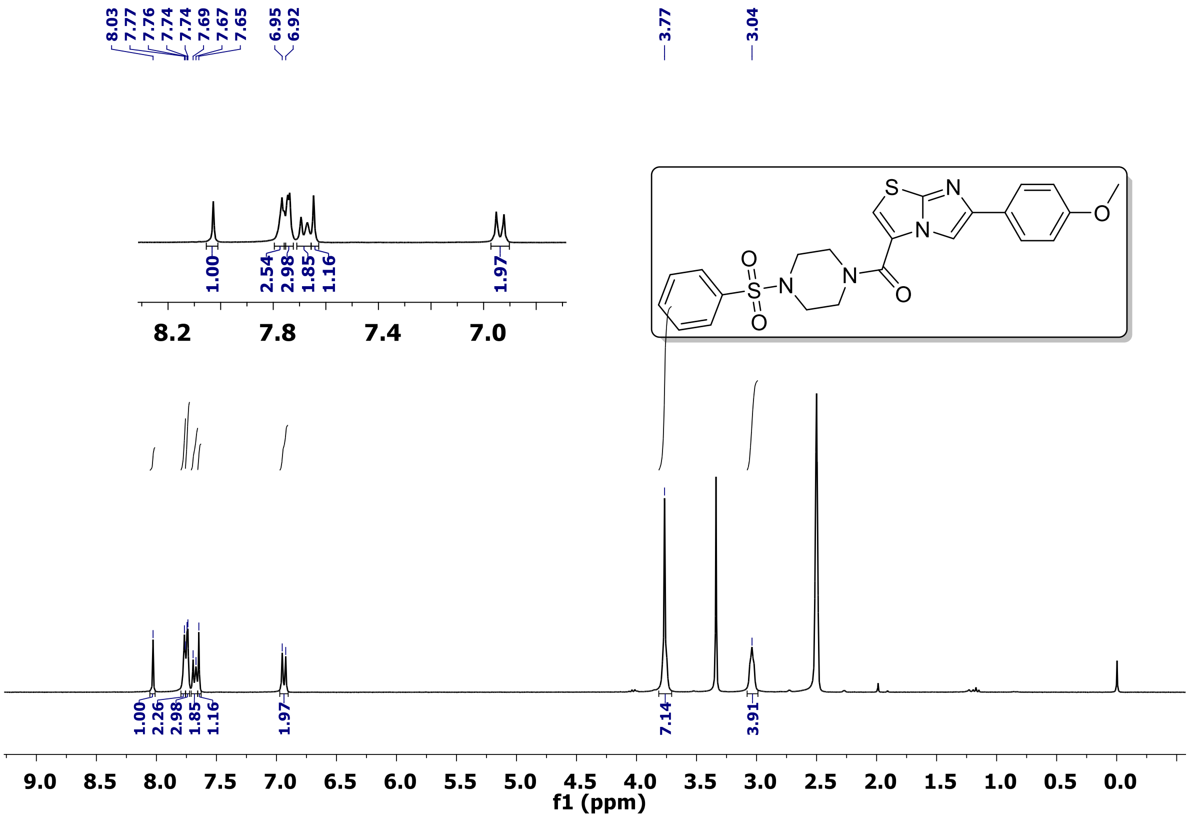

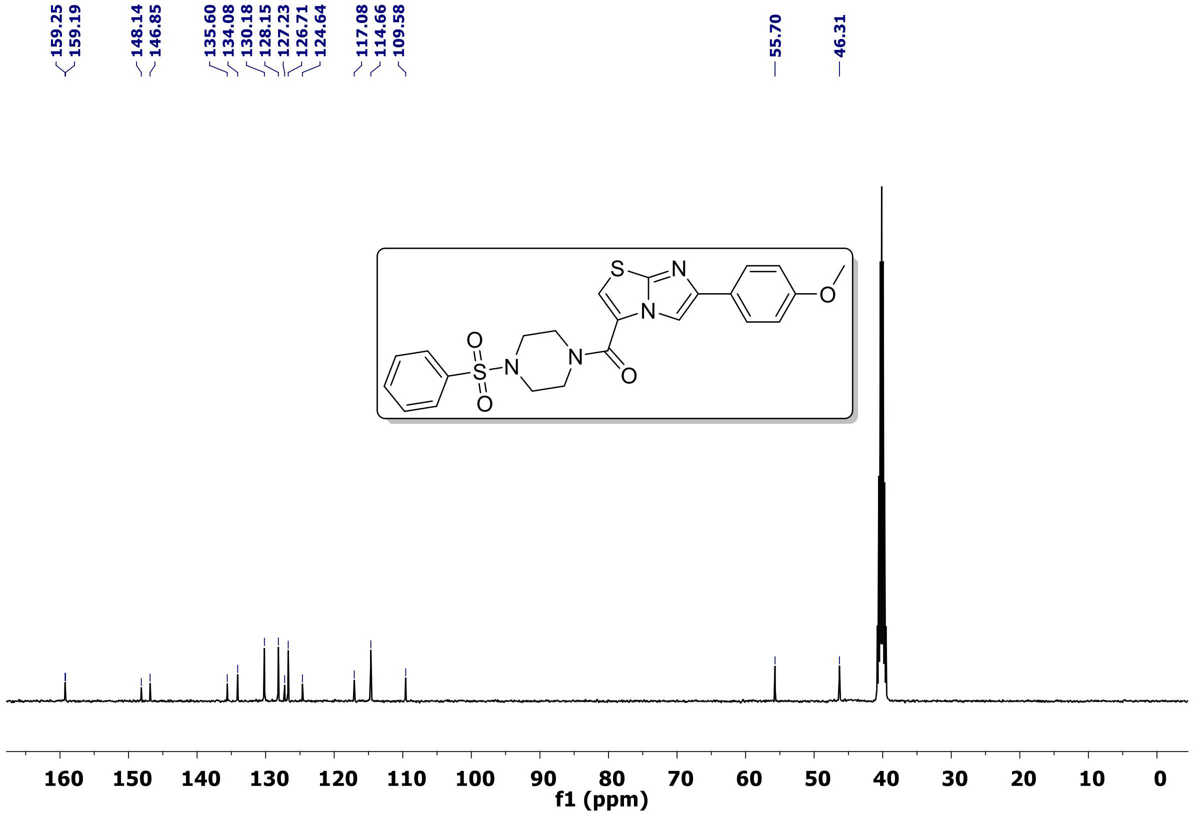

Copy of ^{1}H NMR and ^{13}C NMR spectra of **9ae**

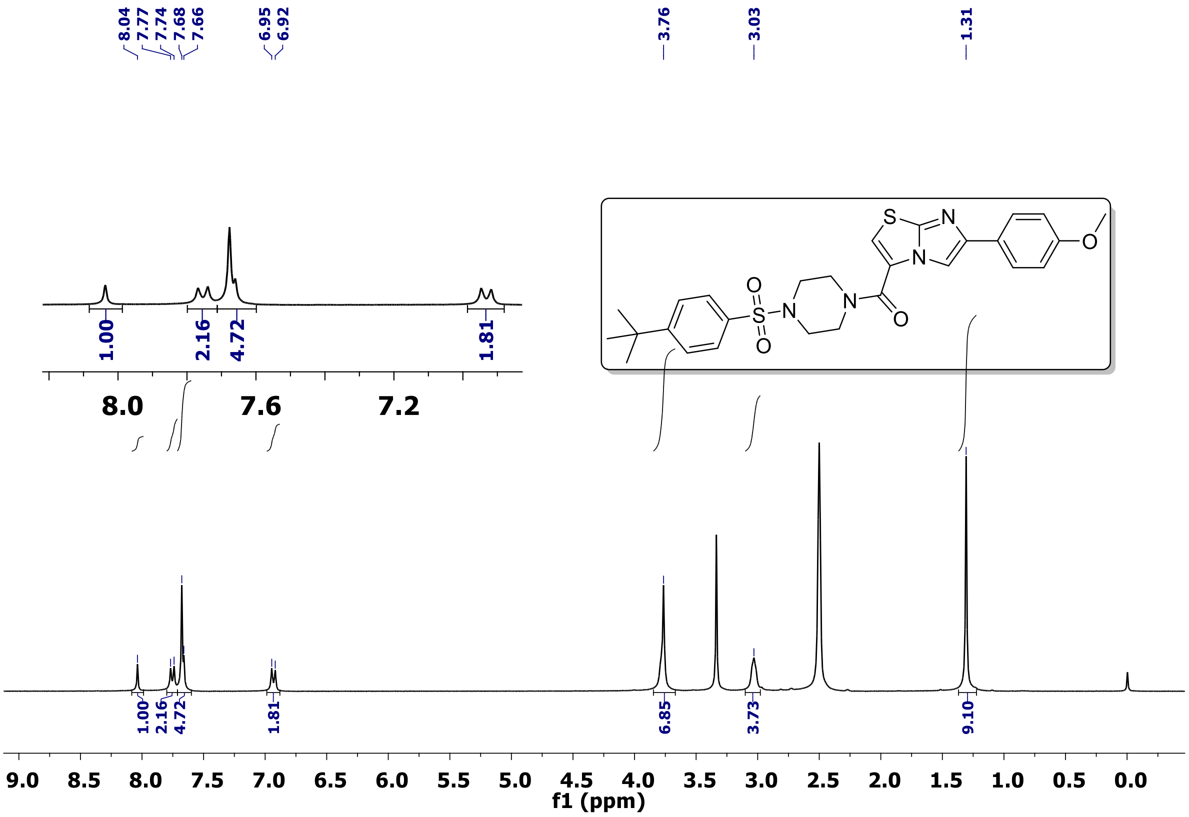

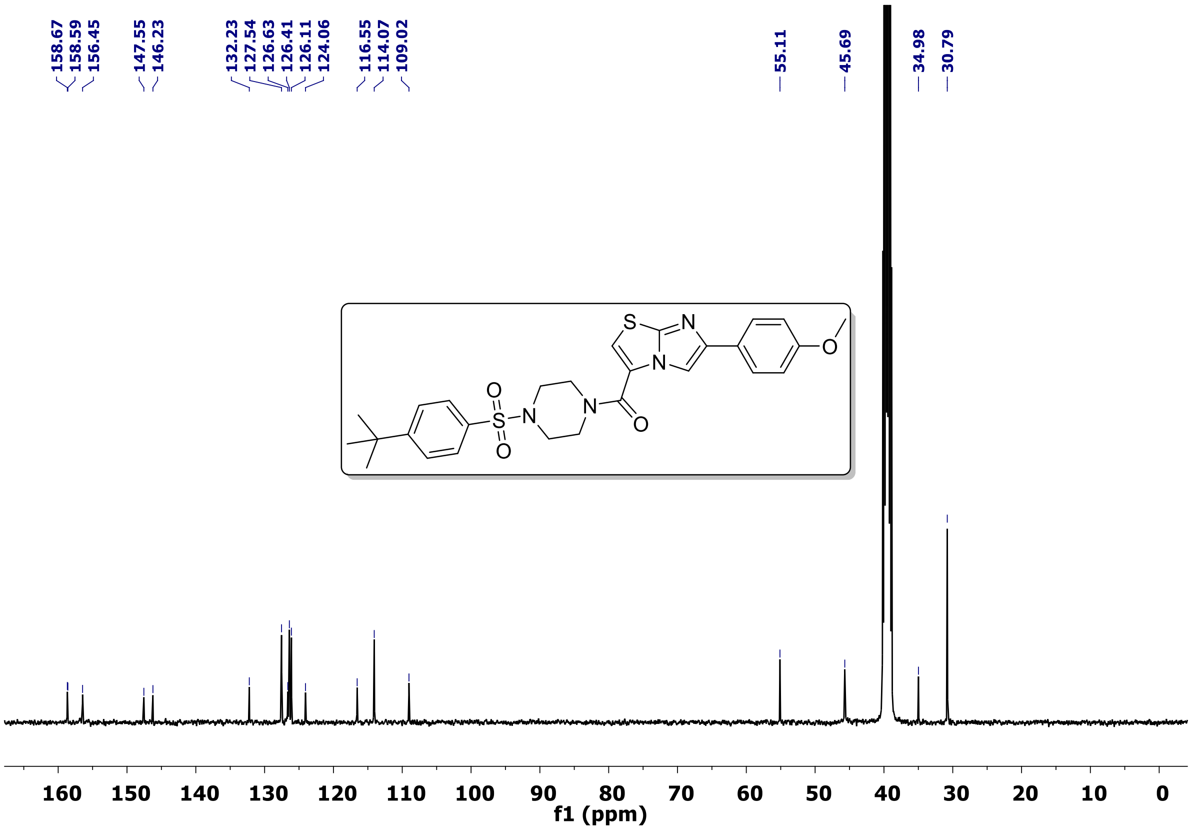

Copy of ^{1}H NMR and ^{13}C NMR spectra of **9ba**

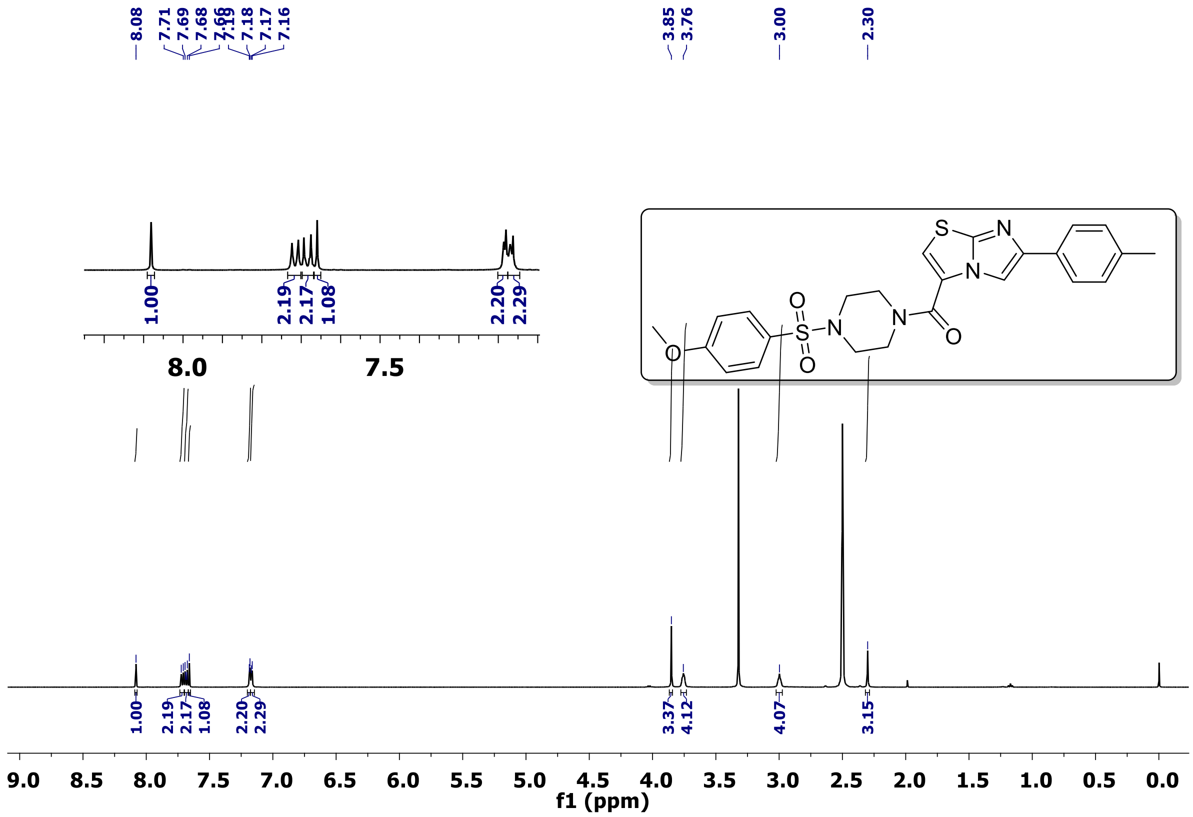

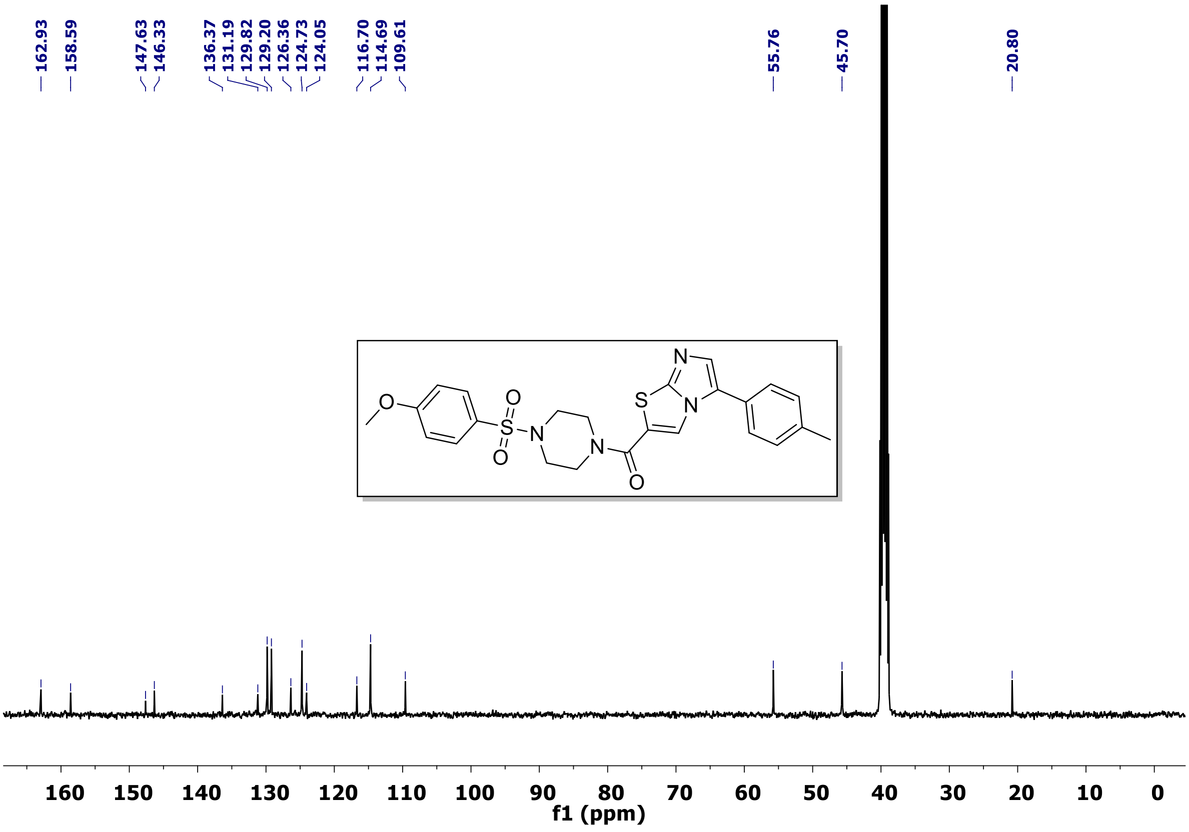

Copy of ^{1}H NMR and ^{13}C NMR spectra of **9bb**

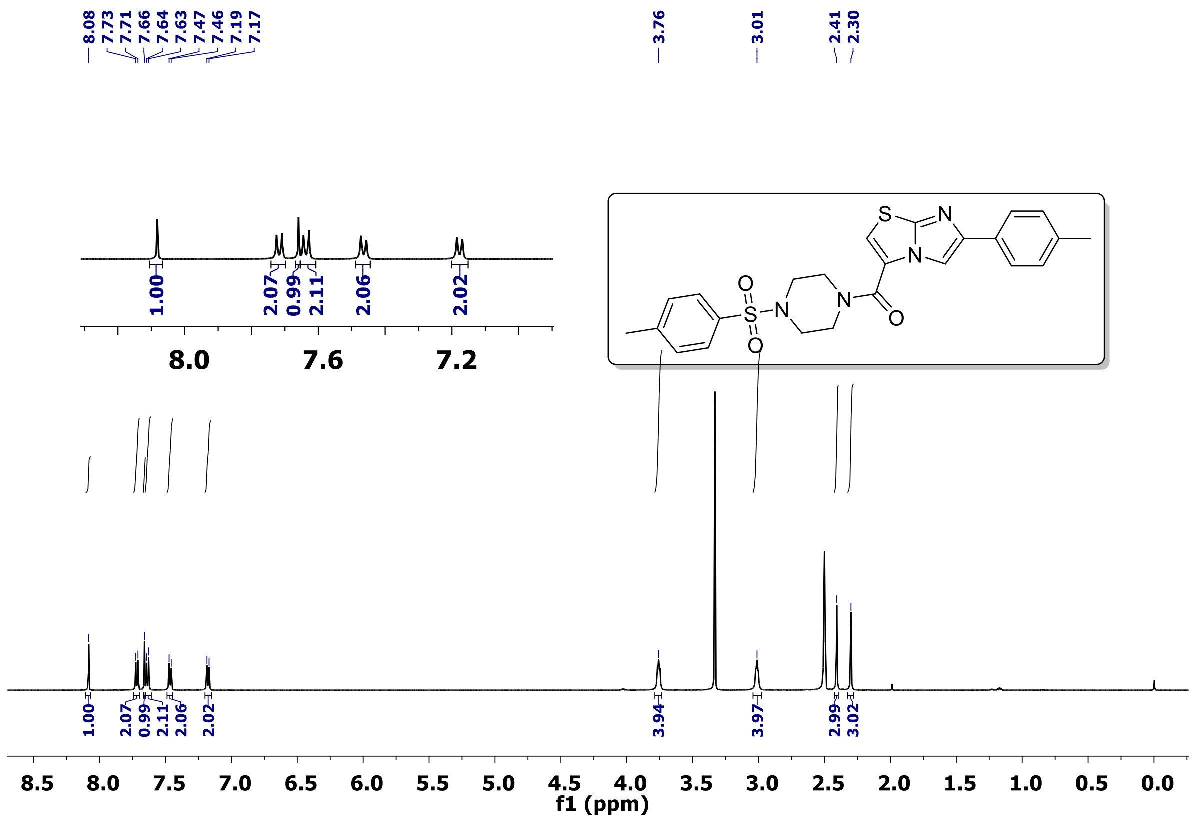

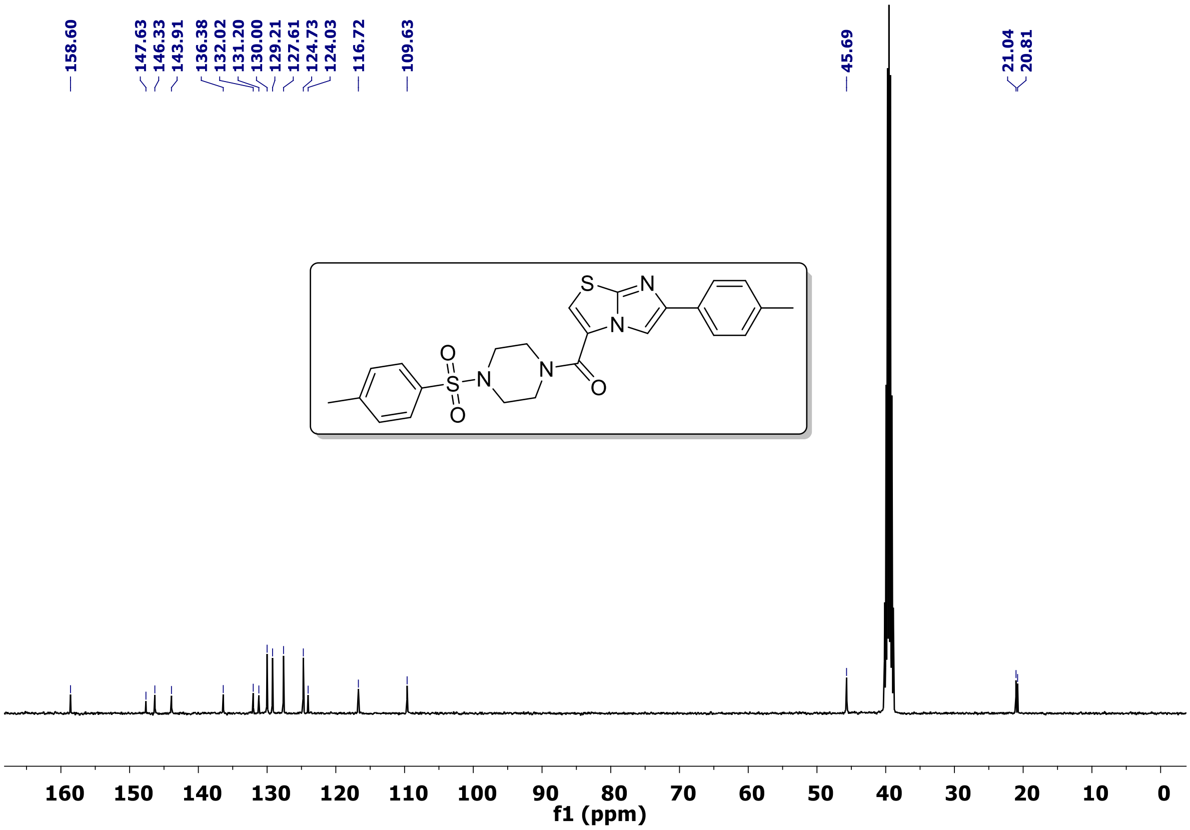

Copy of ^{1}H NMR and ^{13}C NMR spectra of **9bc**

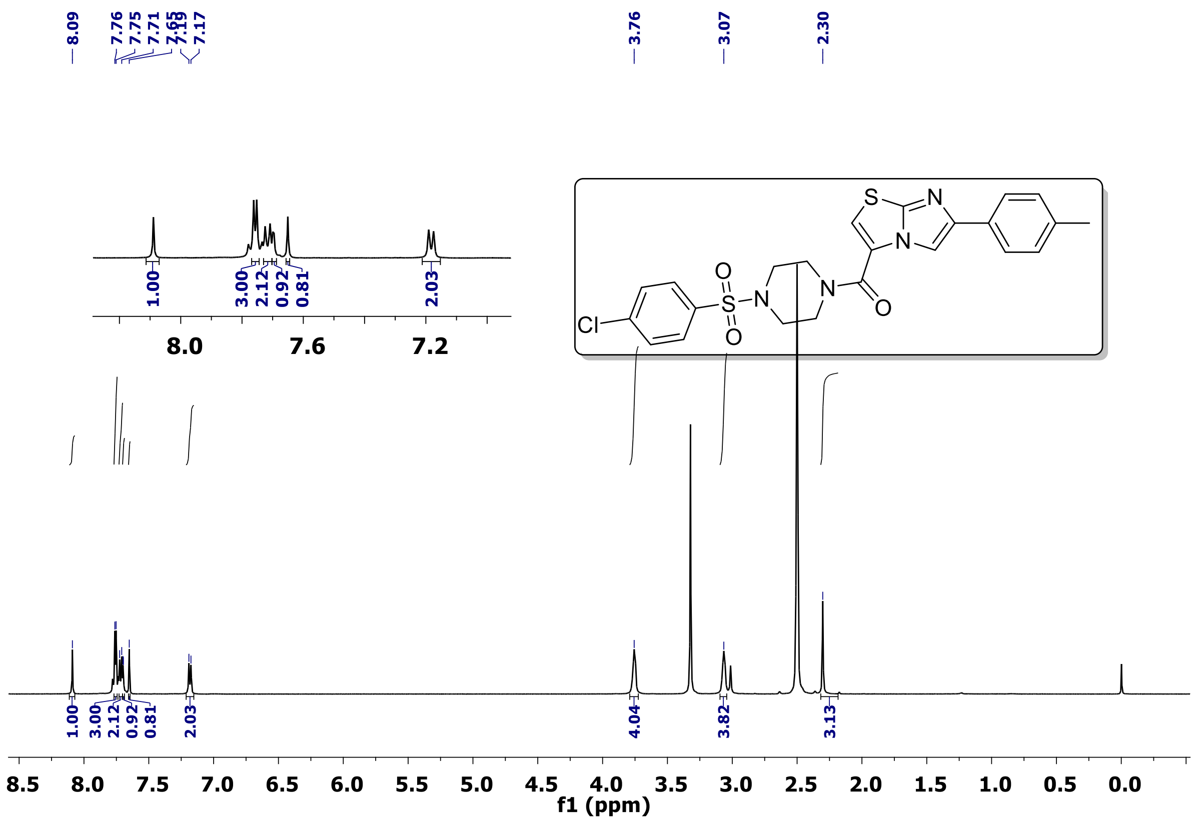

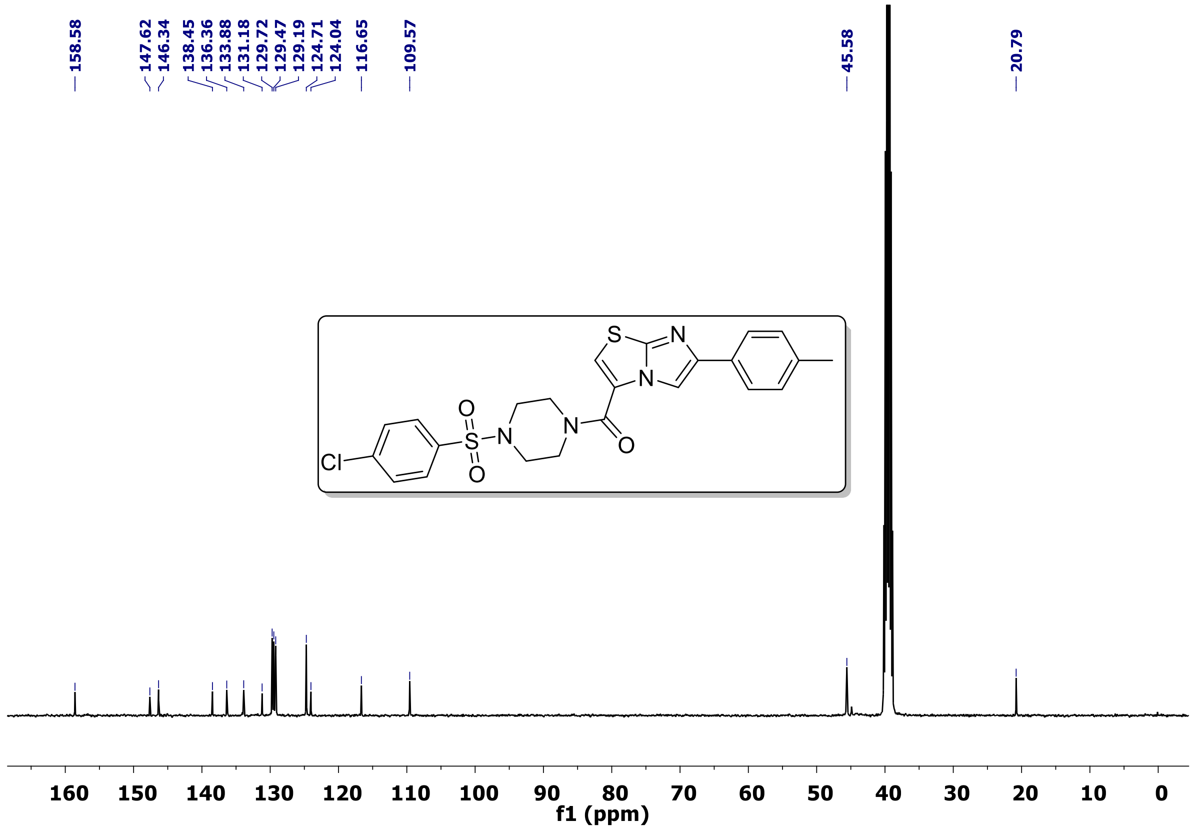

Copy of ^{1}H NMR and ^{13}C NMR spectra of **9bd**

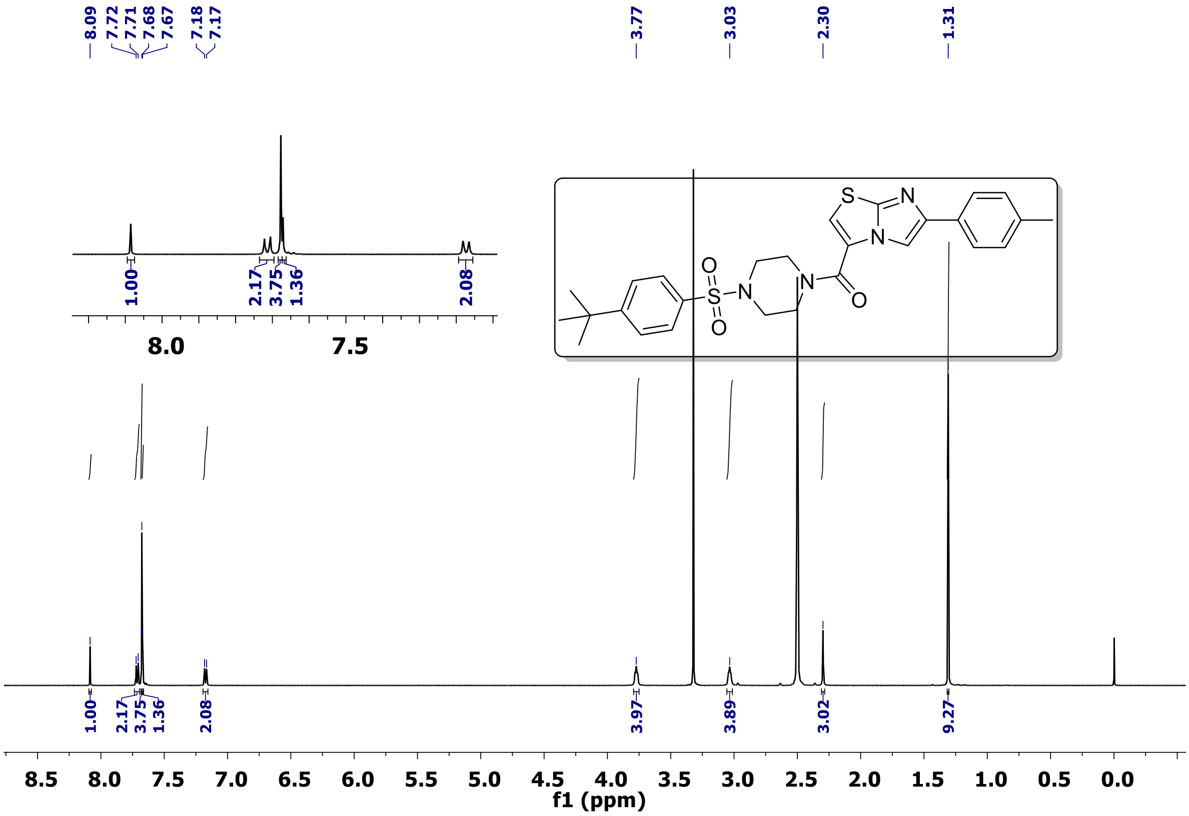

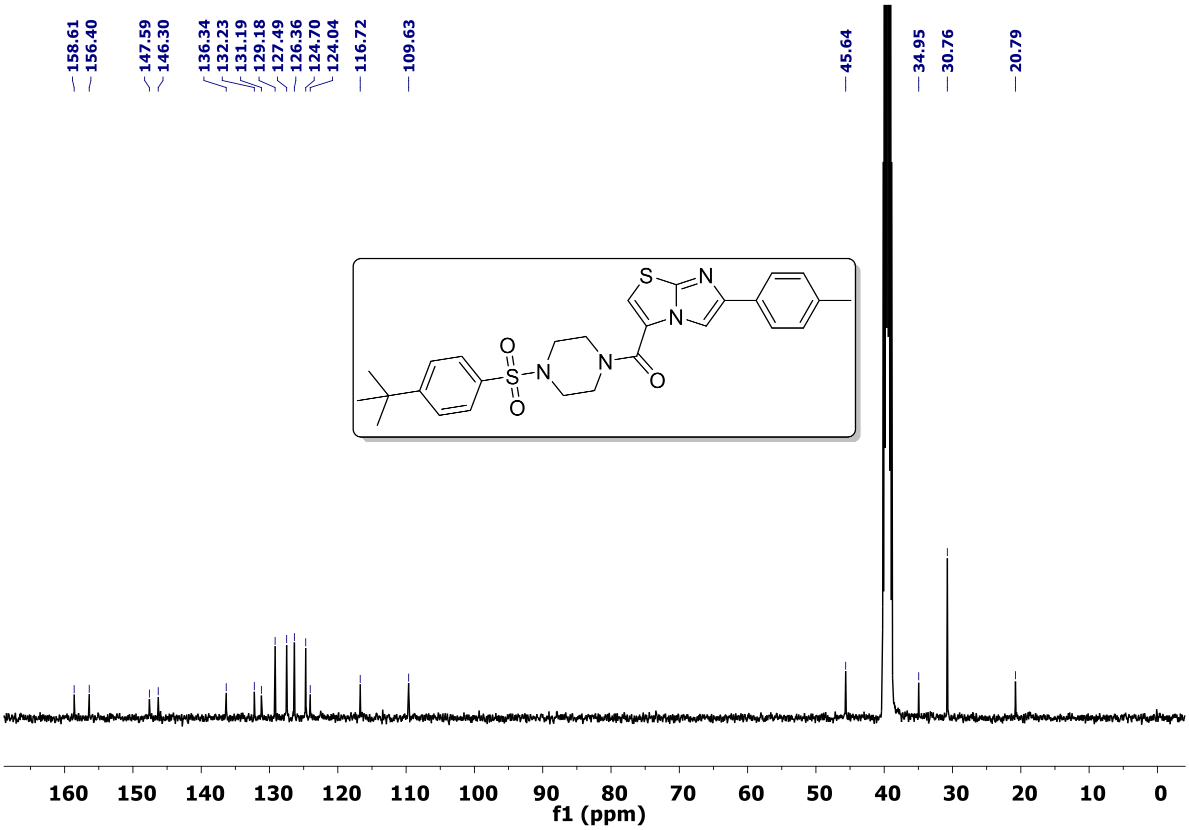

Copy of ^{1}H NMR and ^{13}C NMR spectra of **9ca**

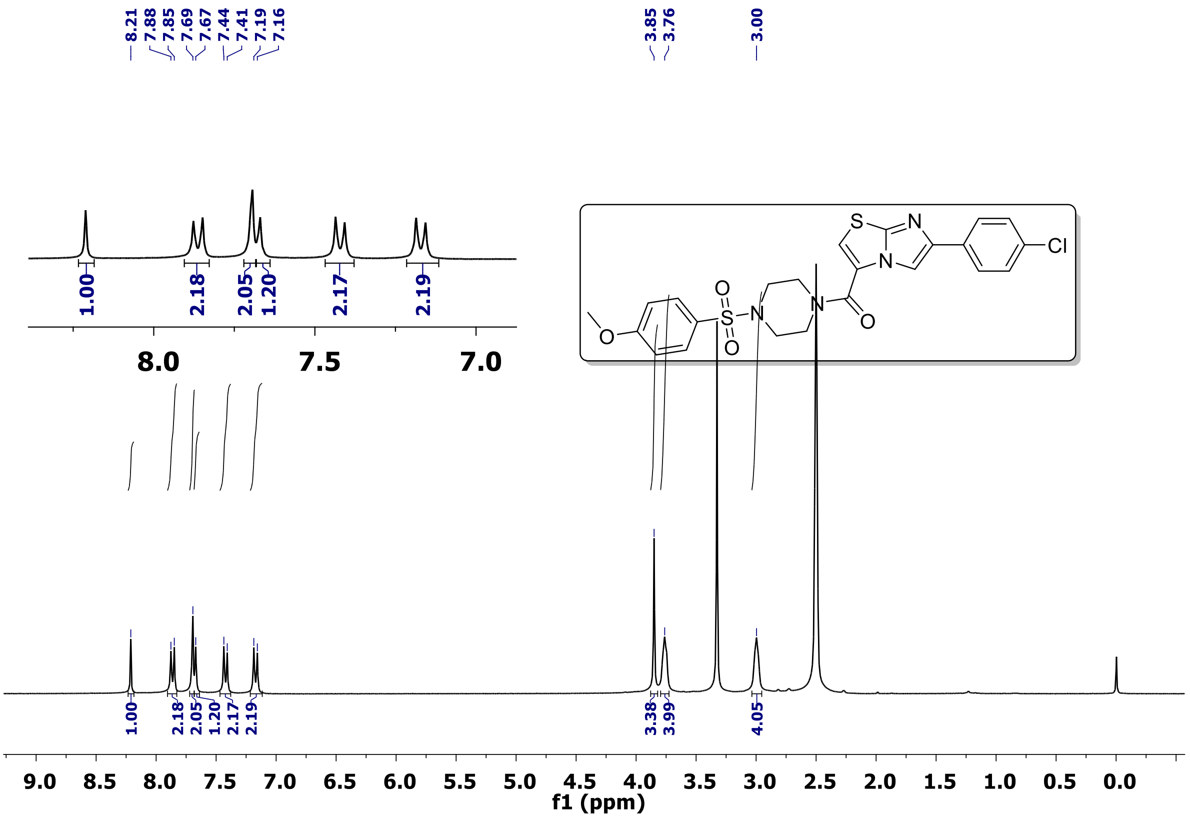

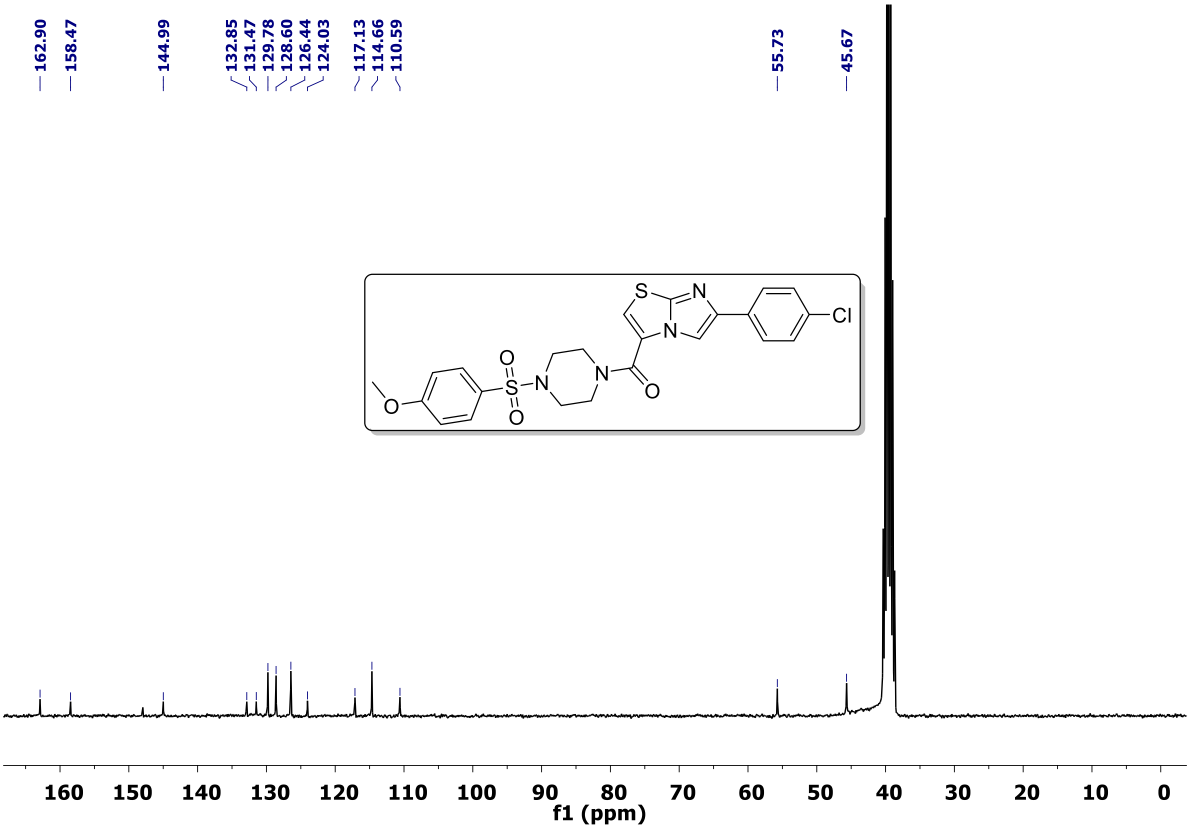

Copy of ^{1}H NMR and ^{13}C NMR spectra of **9cb**

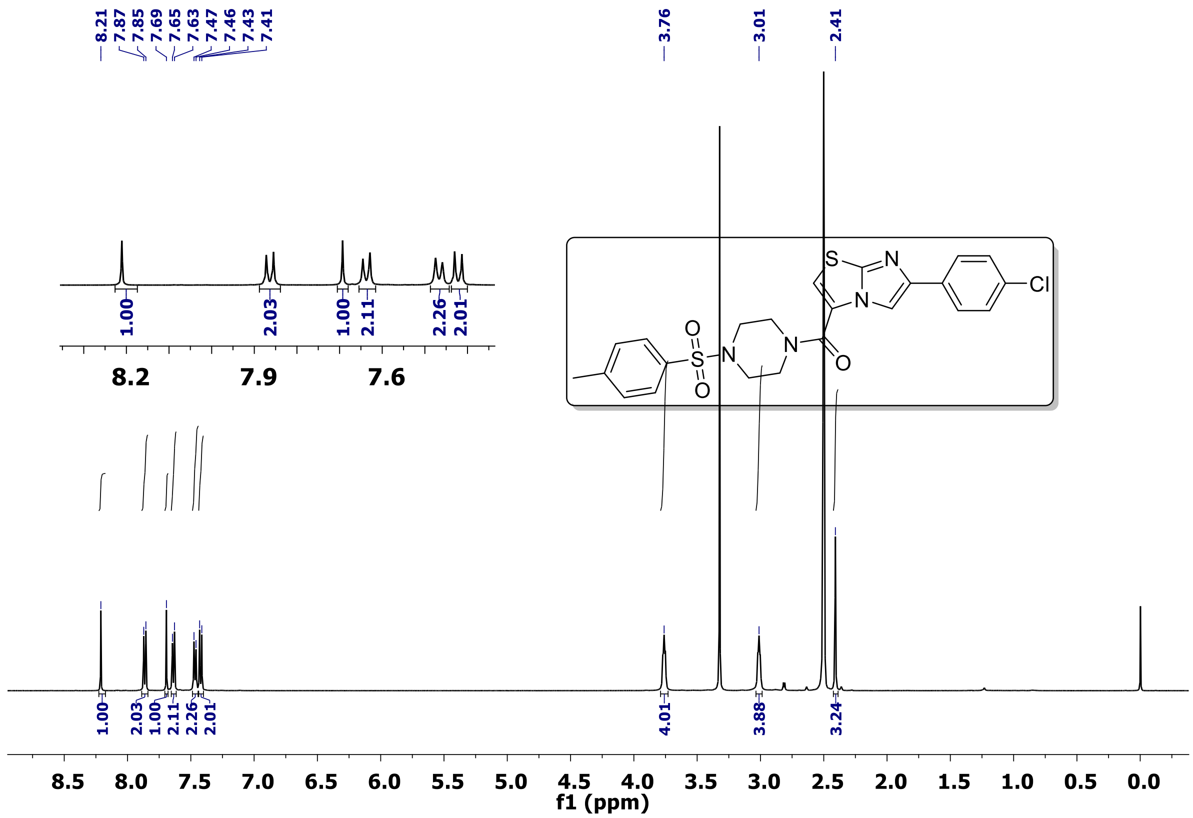

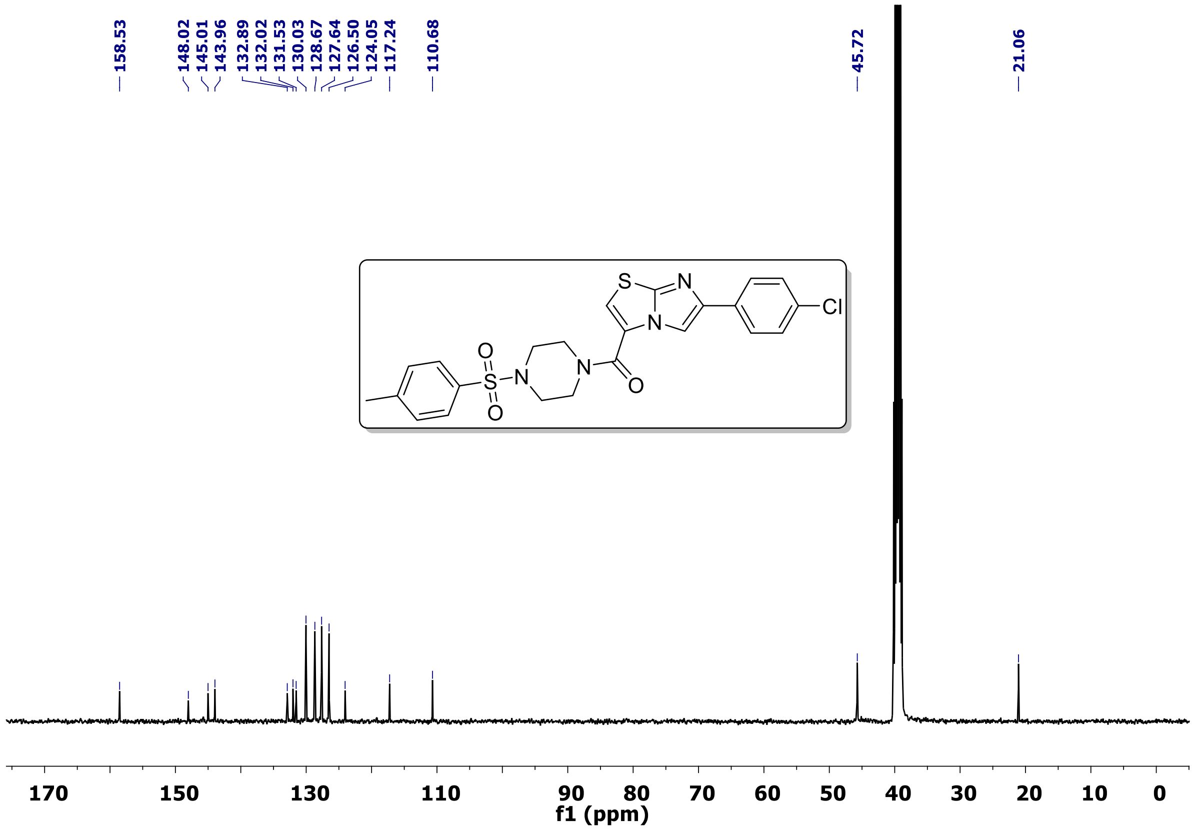

Copy of ^{1}H NMR and ^{13}C NMR spectra of **9cc**

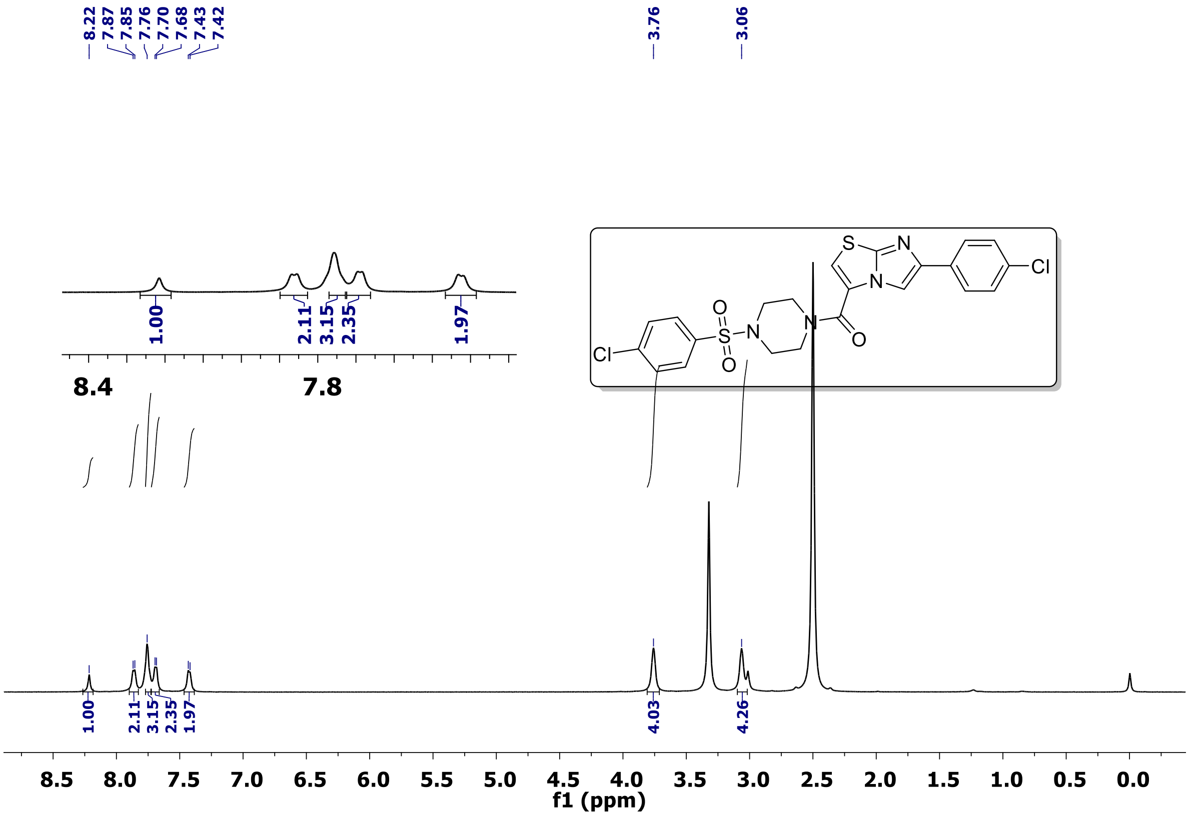

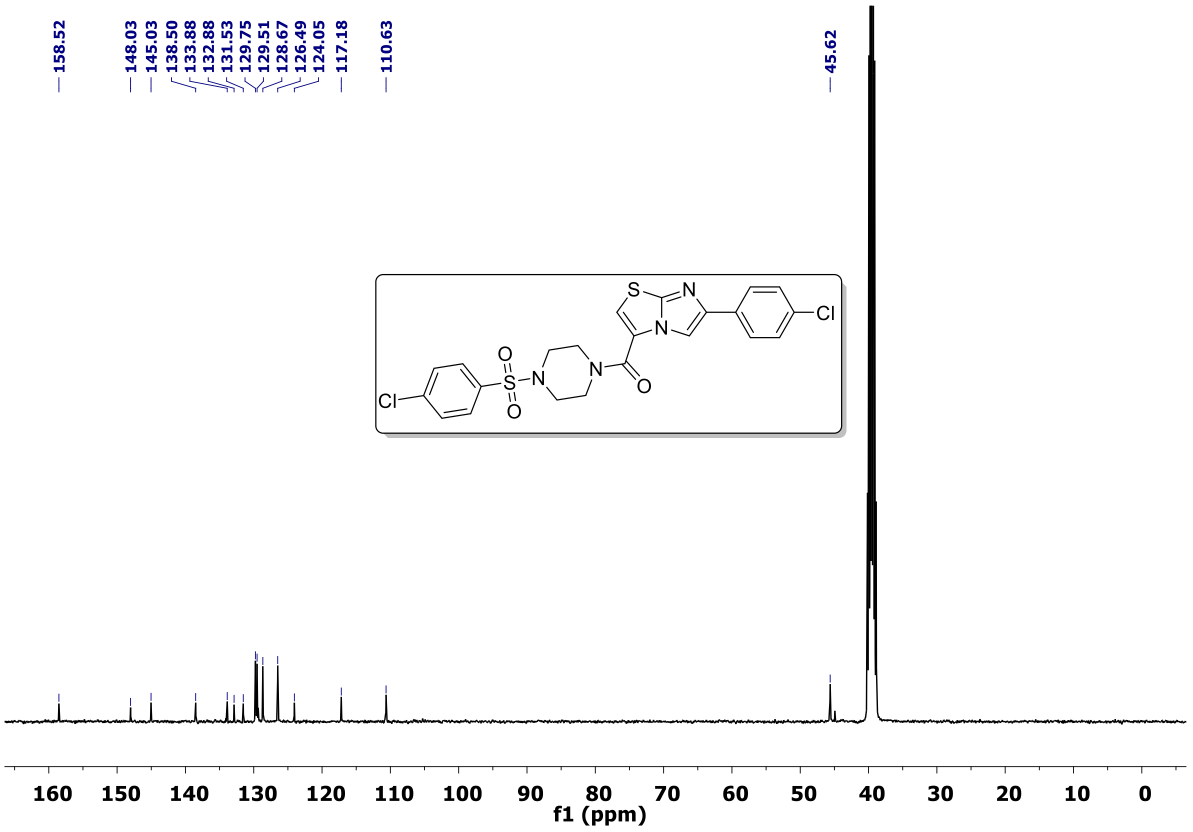

Copy of ^{1}H NMR and ^{13}C NMR spectra of **9cd**

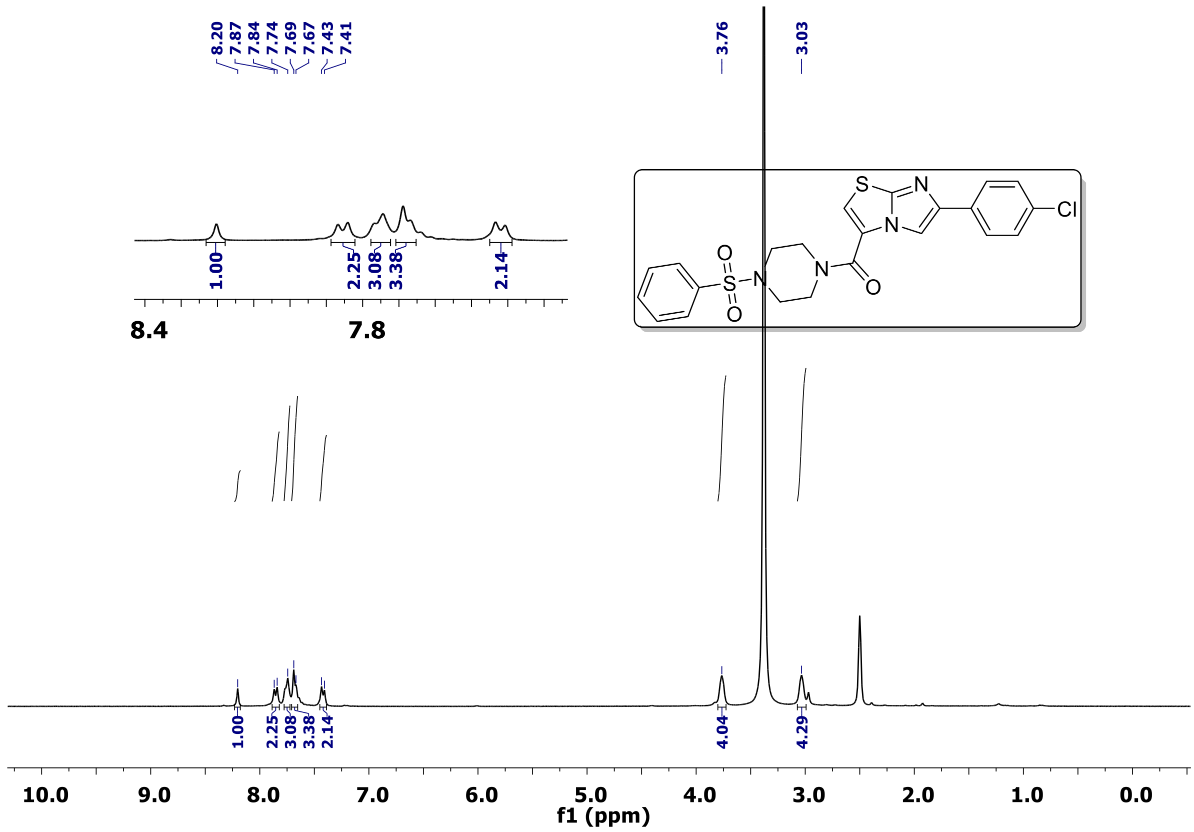

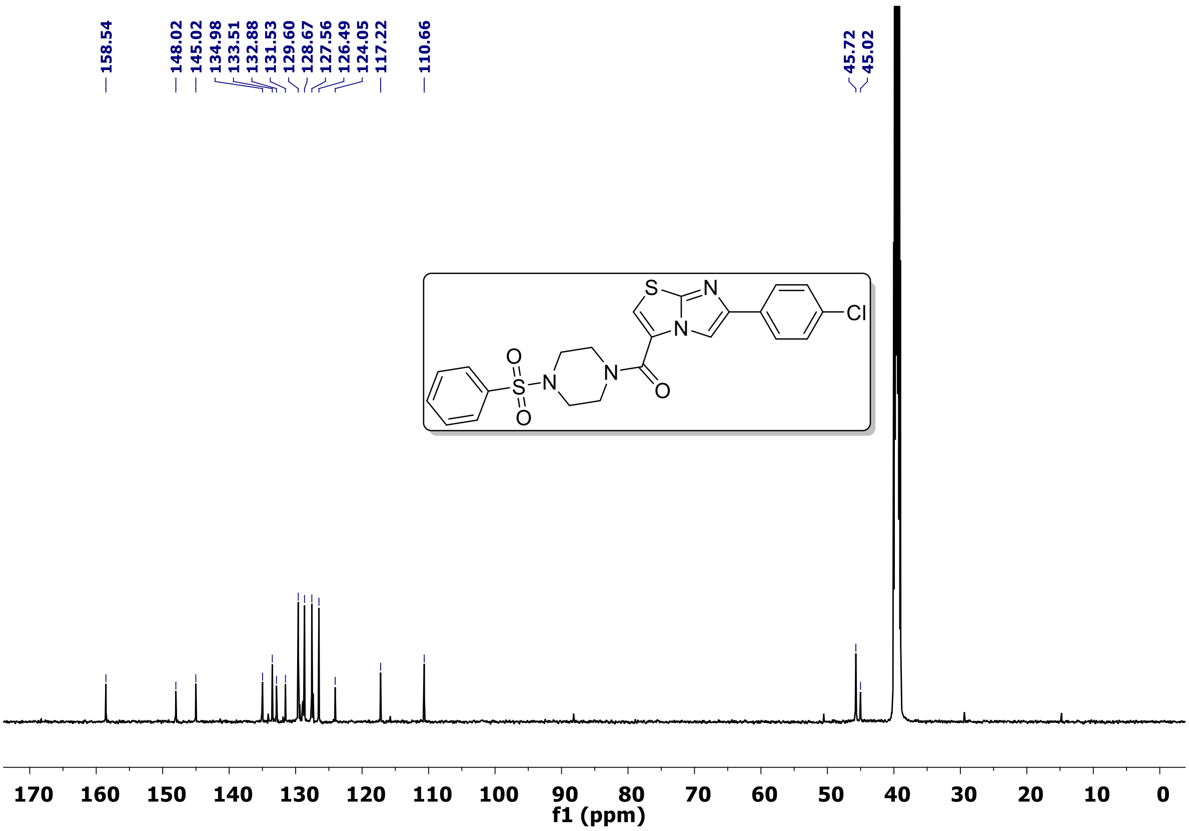

Copy of ^{1}H NMR and ^{13}C NMR spectra of**9ce**

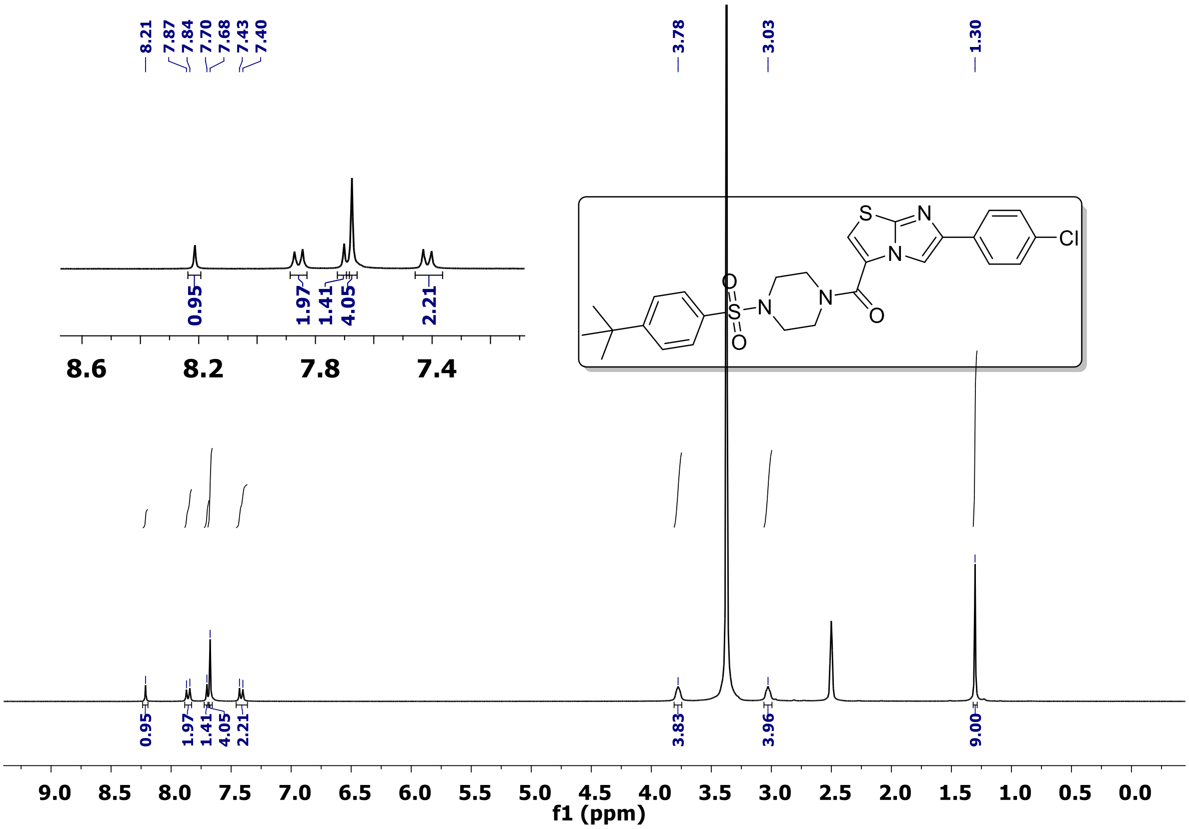

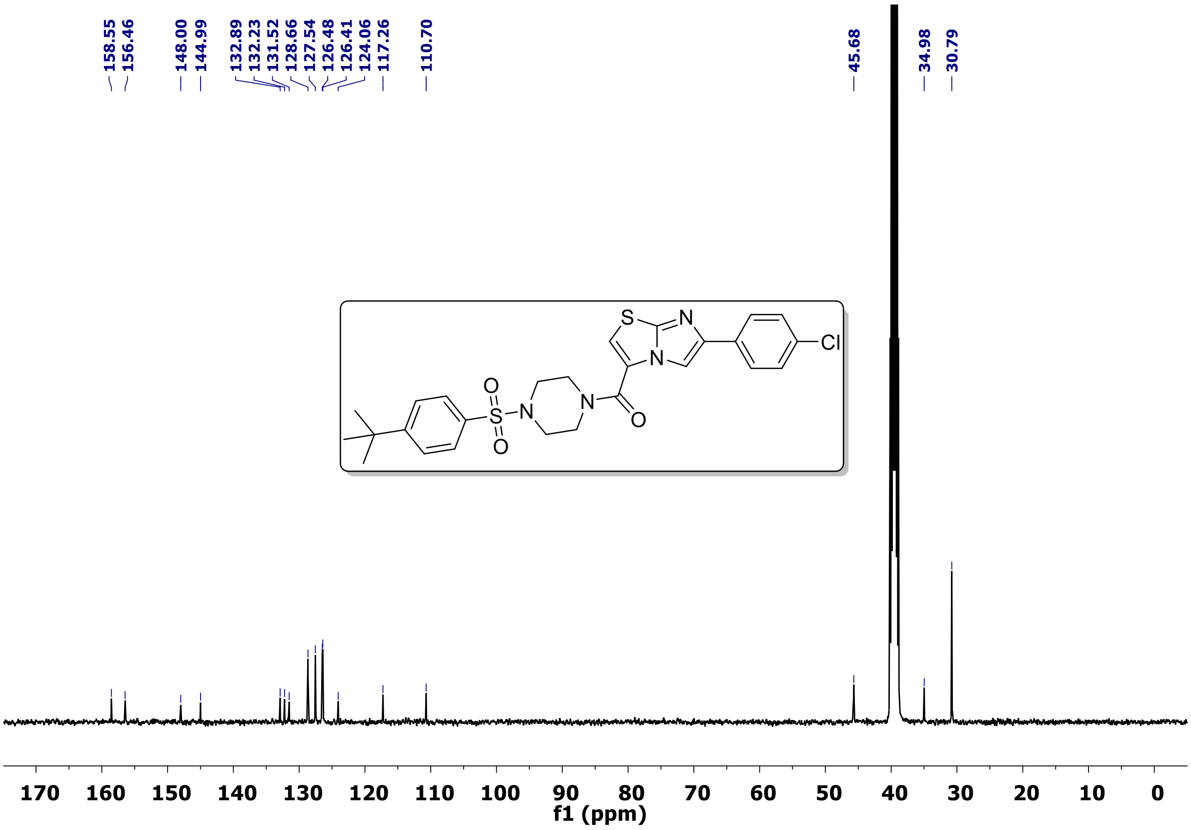

Copy of ^{1}H NMR and ^{13}C NMR spectra of **9da**

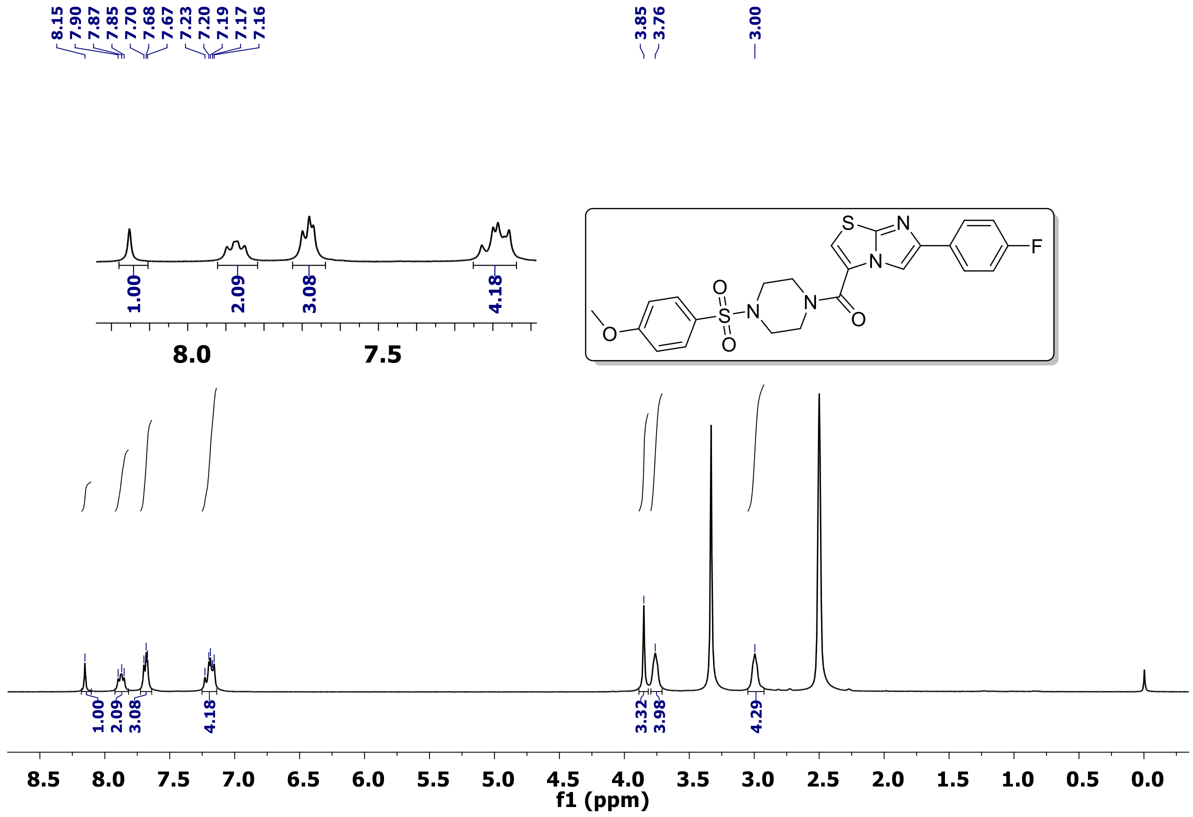

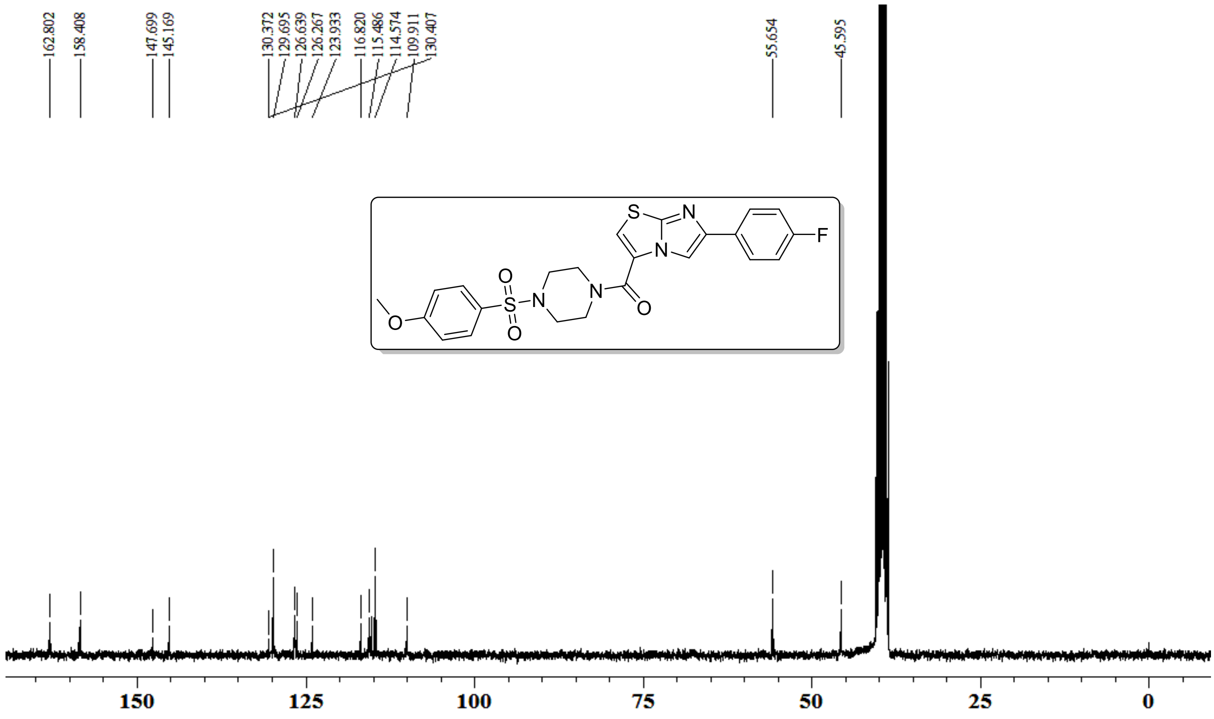

Copy of ^{1}H NMR and ^{13}C NMR spectra of **9db**

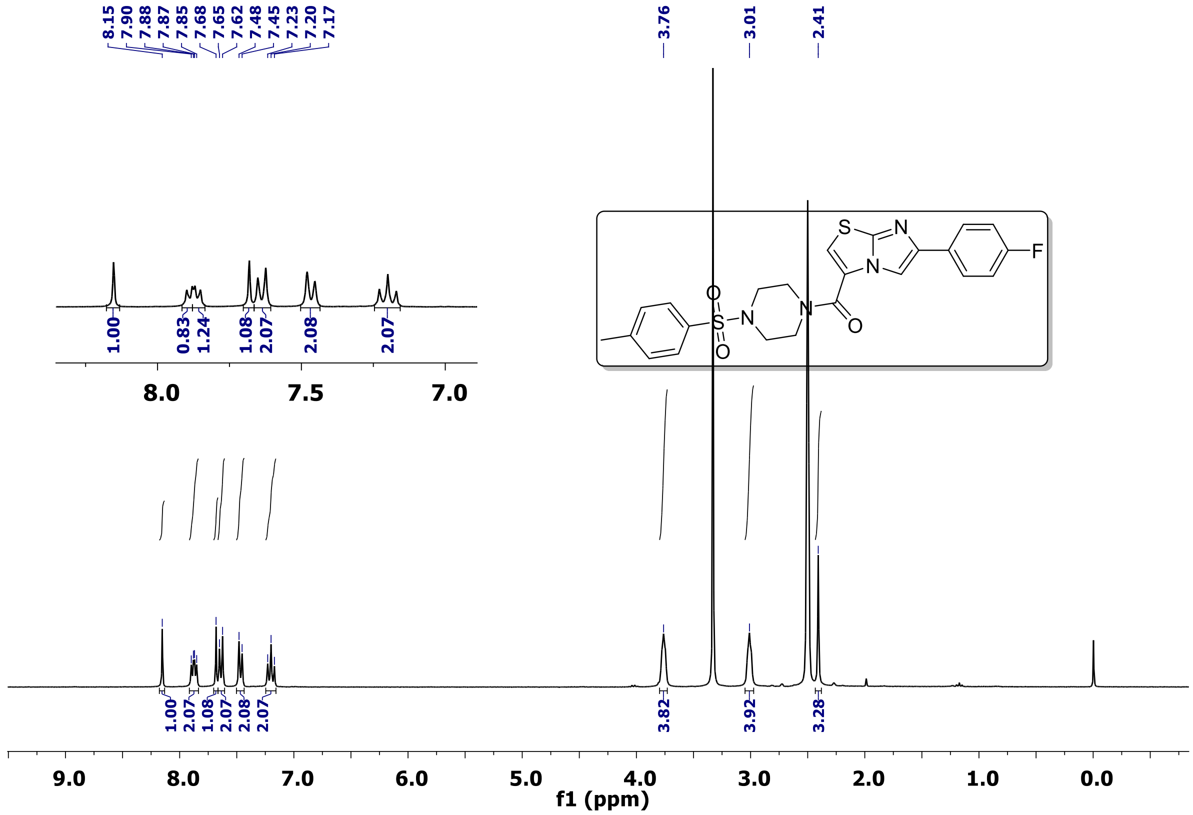

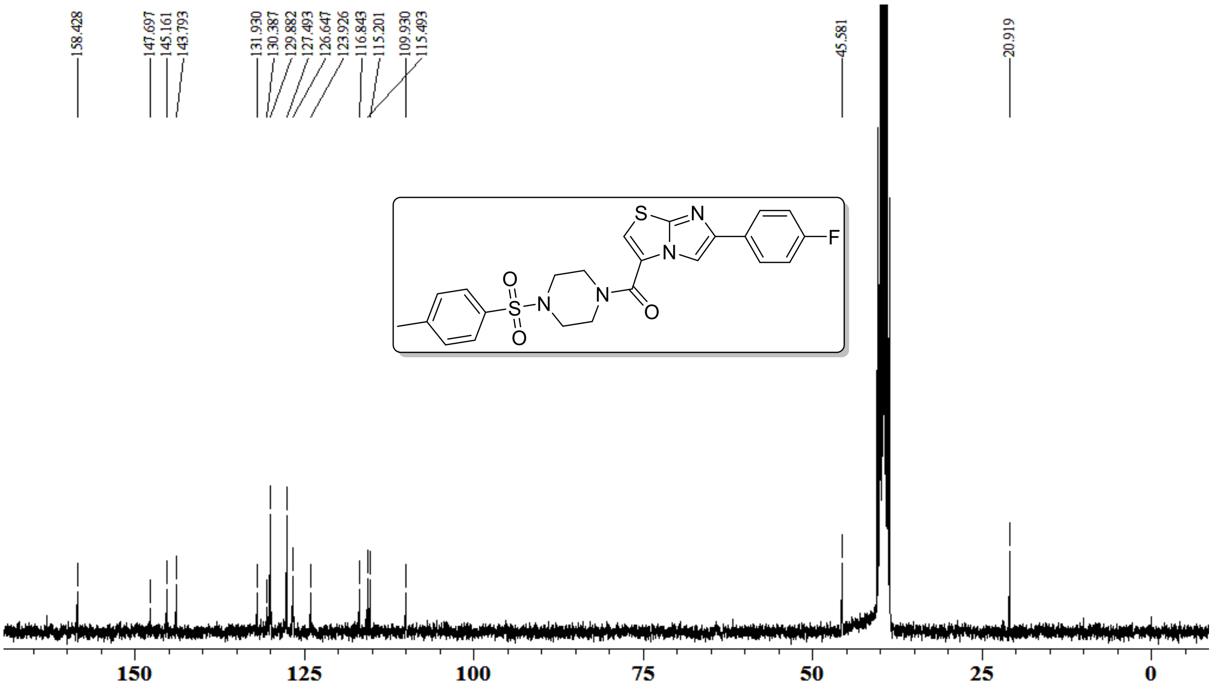

Copy of ^{1}H NMR and ^{13}C NMR spectra of **9dc**

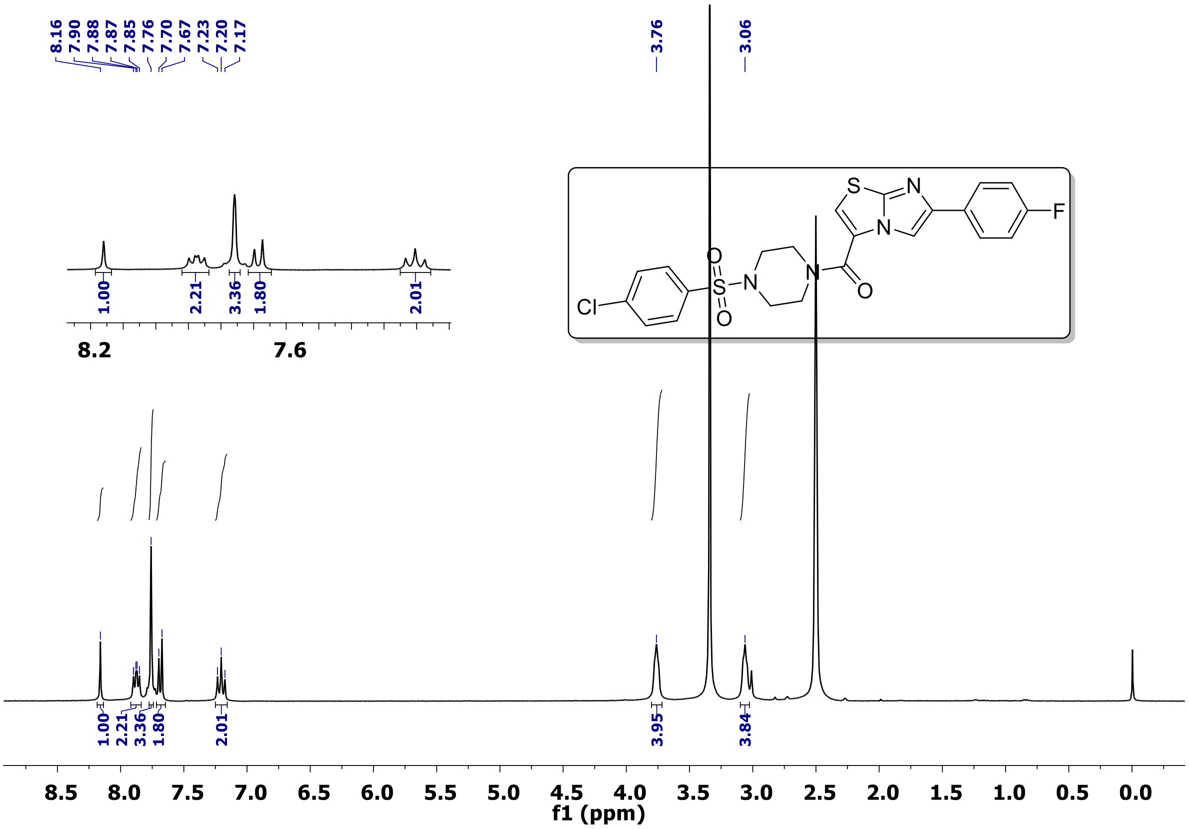

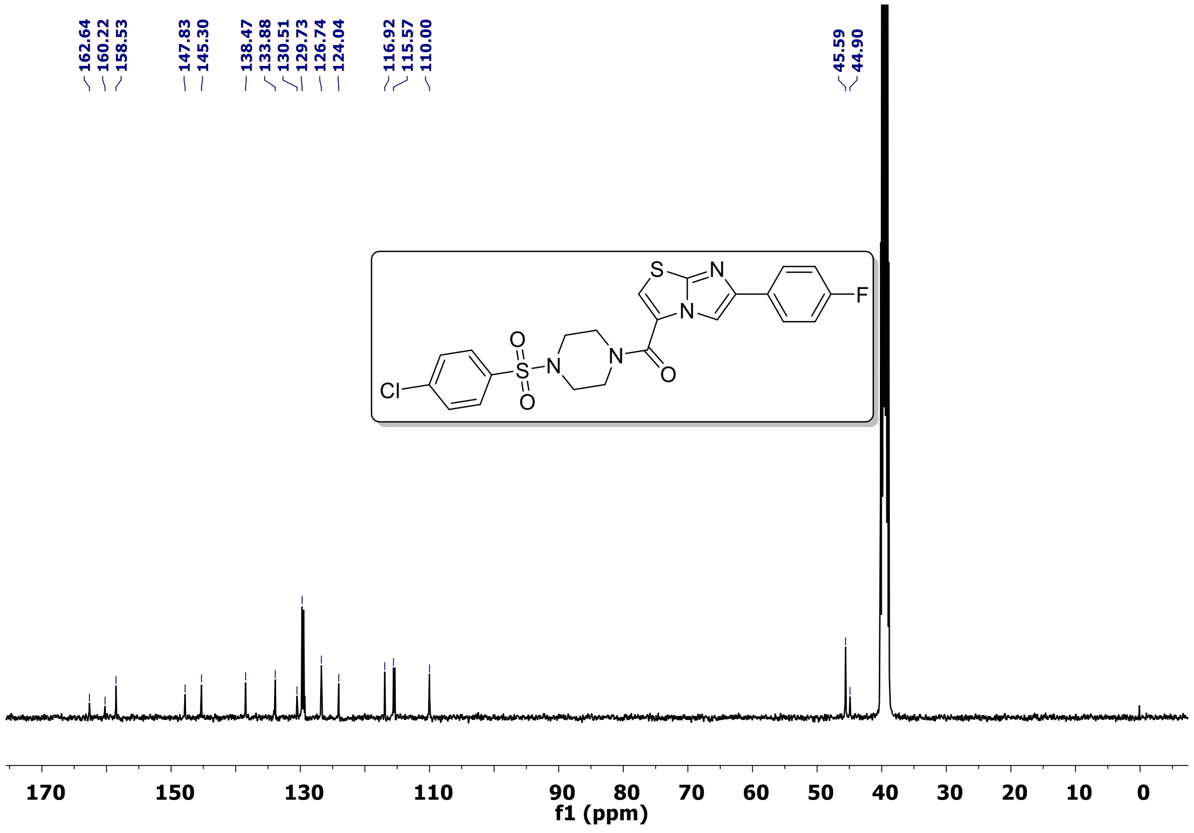

Copy of ^{1}H NMR and ^{13}C NMR spectra of **9dd**

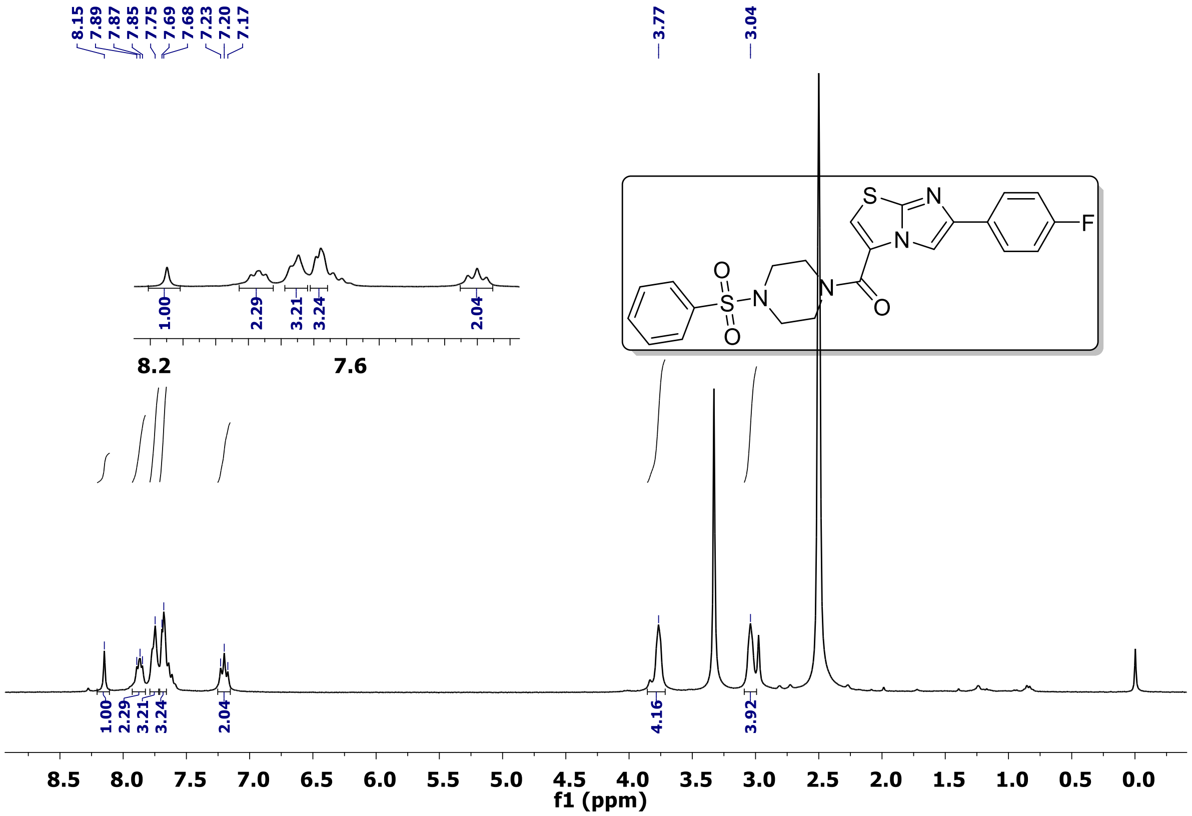

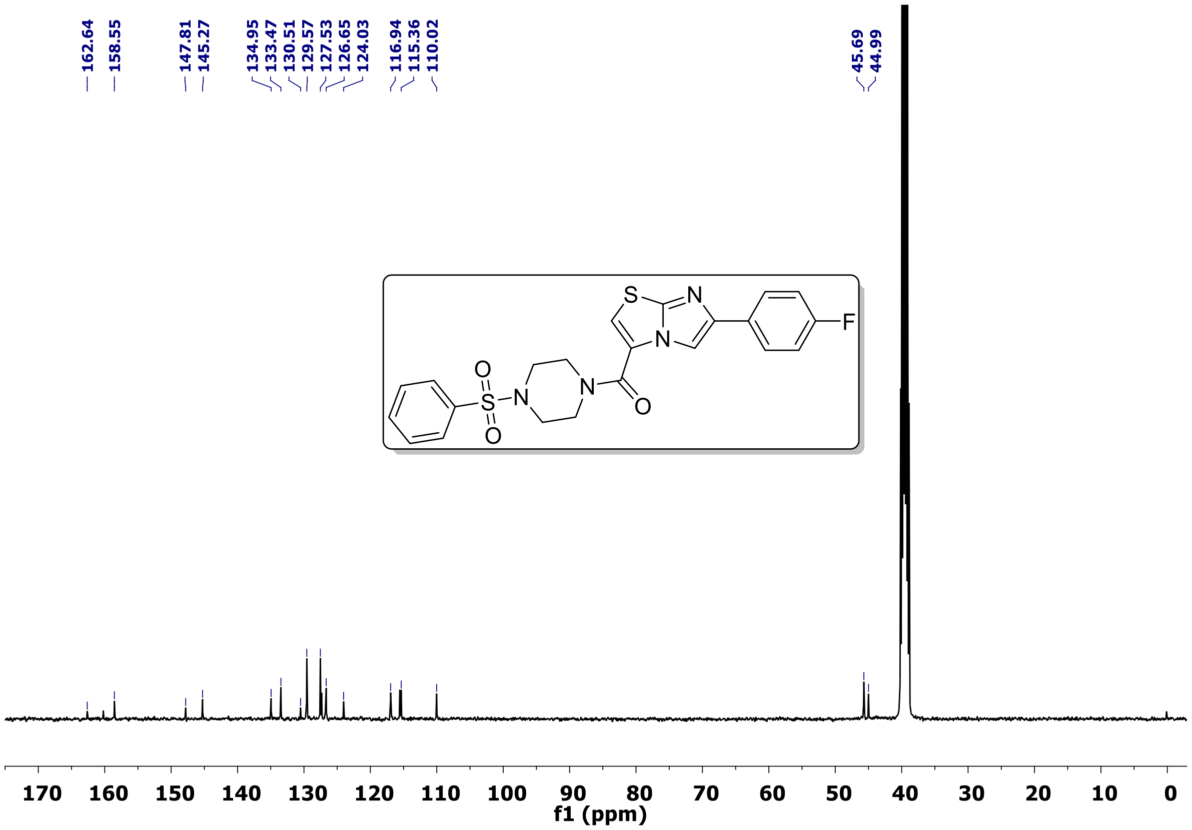

Copy of ^{1}H NMR and ^{13}C NMR spectra of **9de**

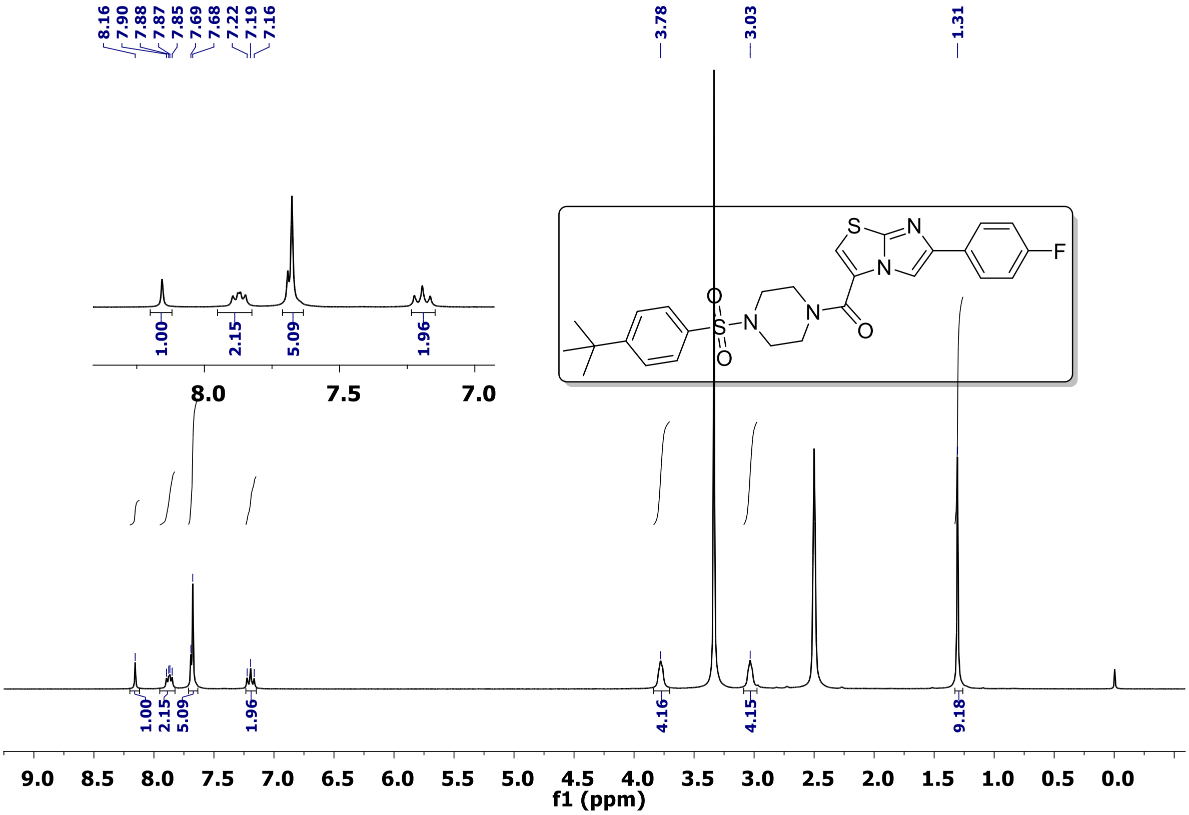

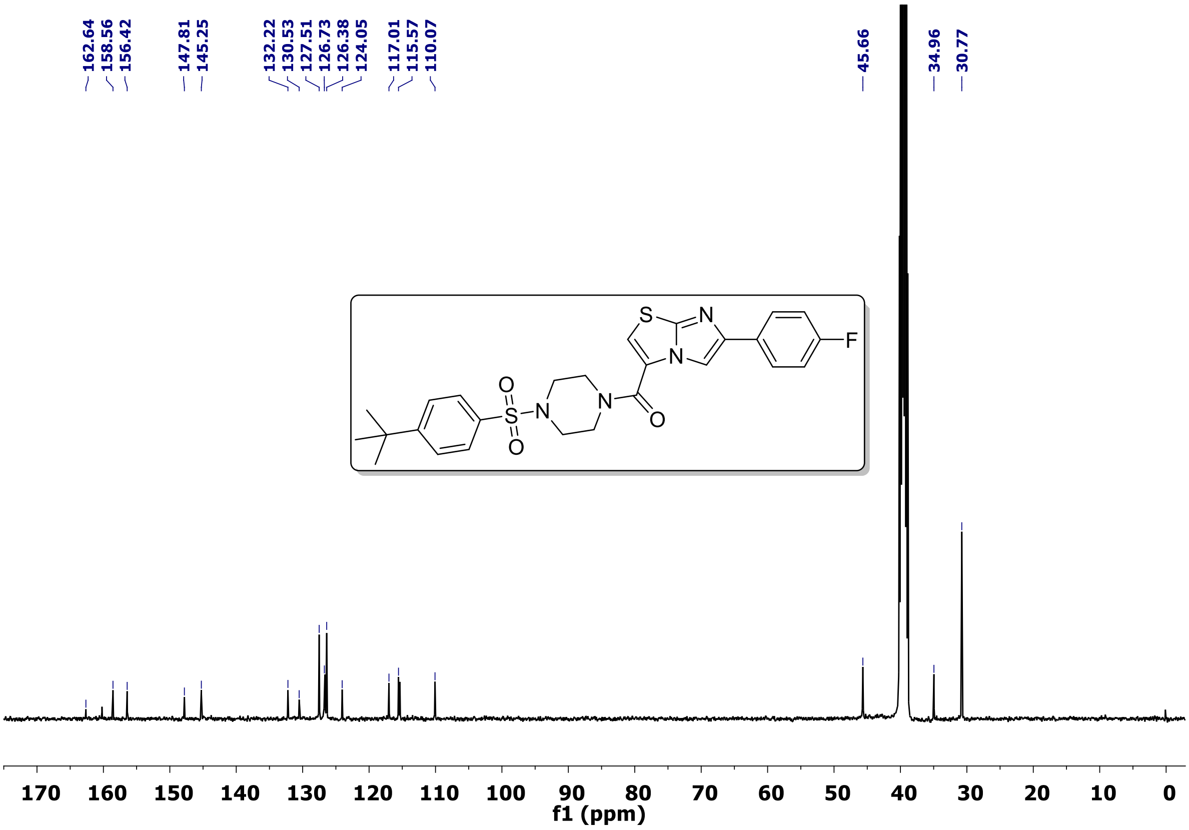

Copy of ^{1}H NMR and ^{13}C NMR spectra of **9ea**

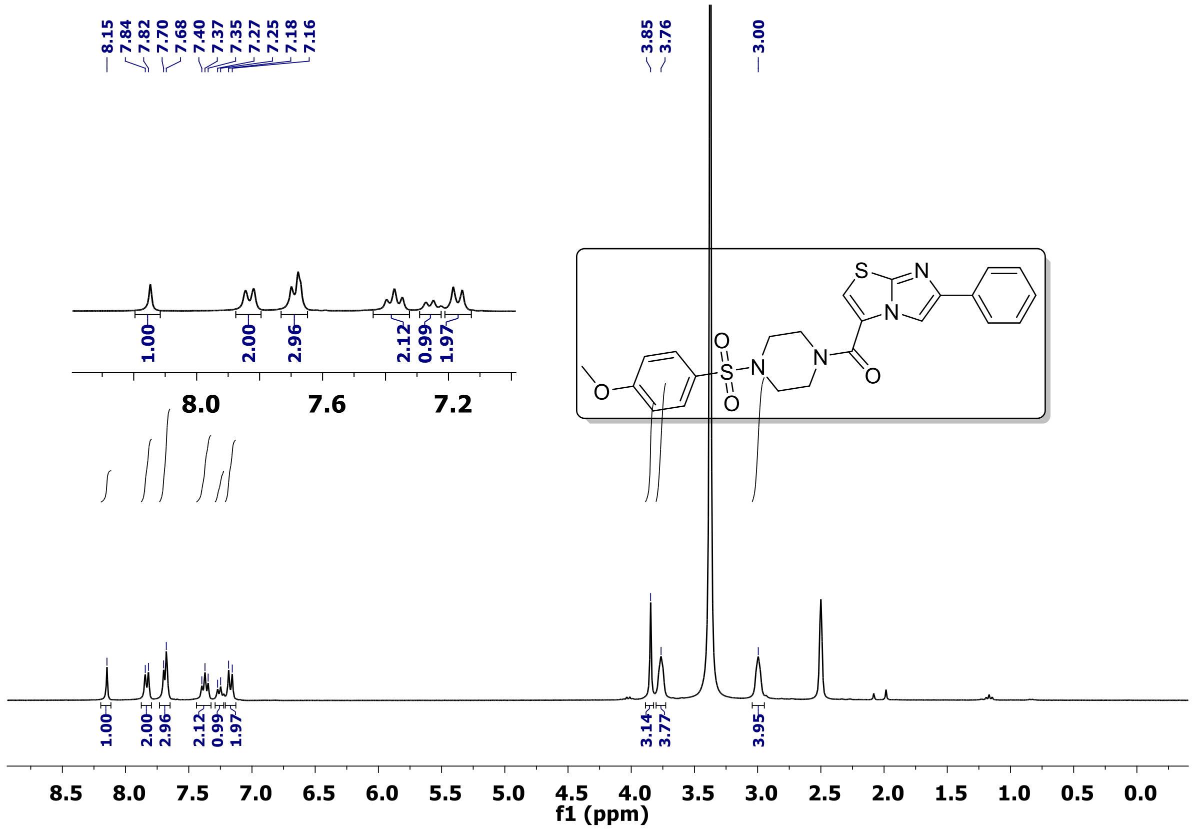

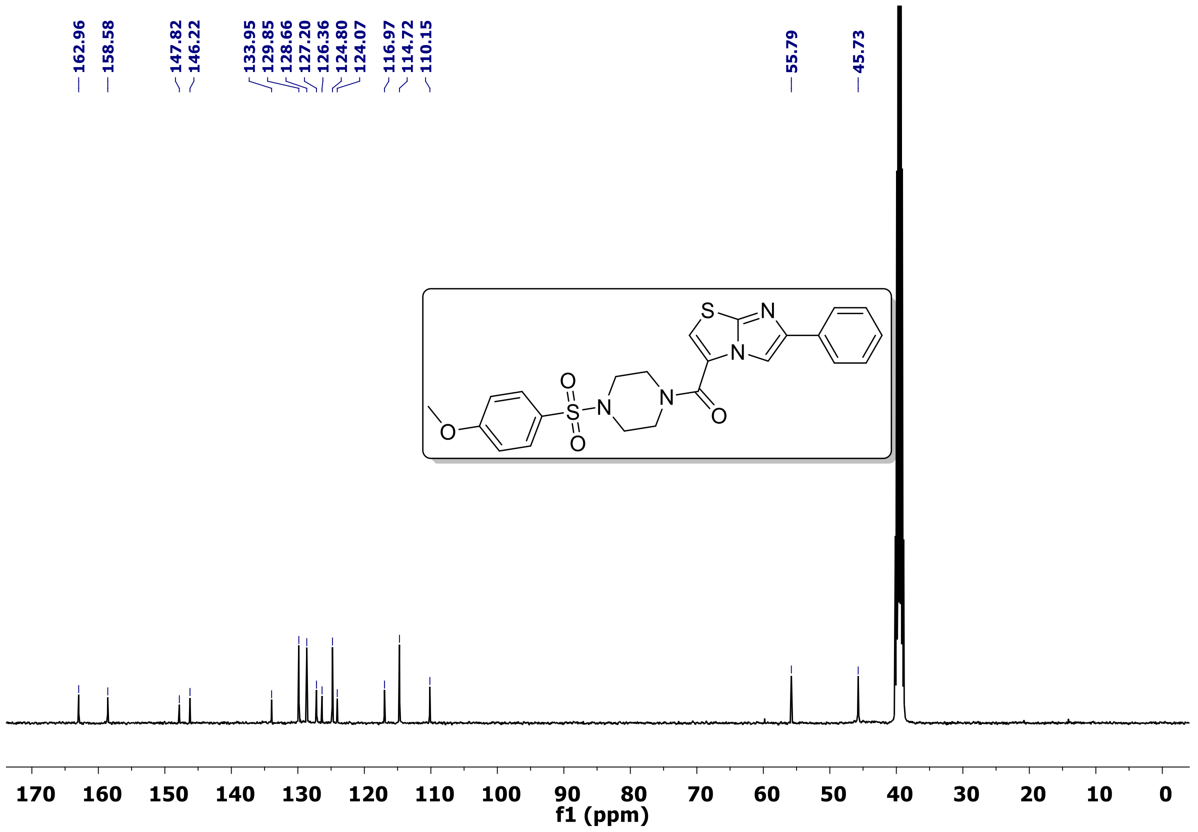

Copy of ^{1}H NMR and ^{13}C NMR spectra of **9eb**

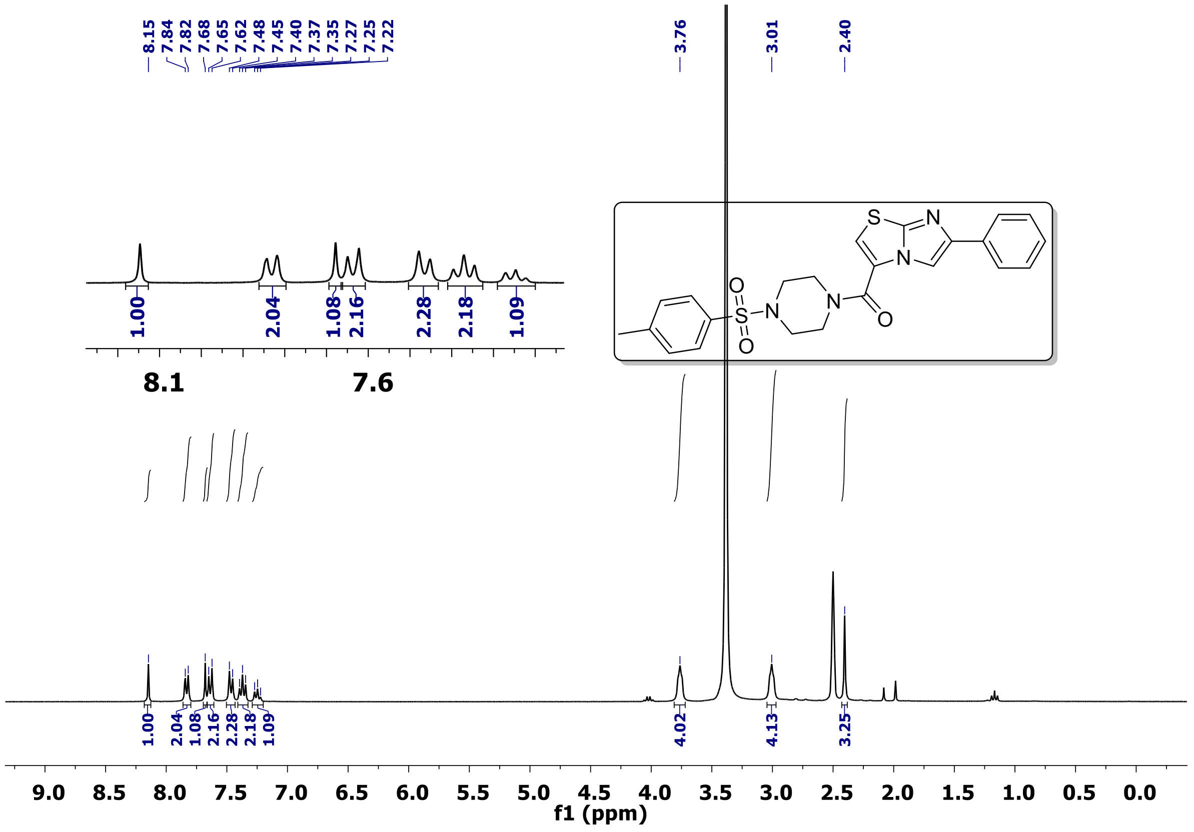

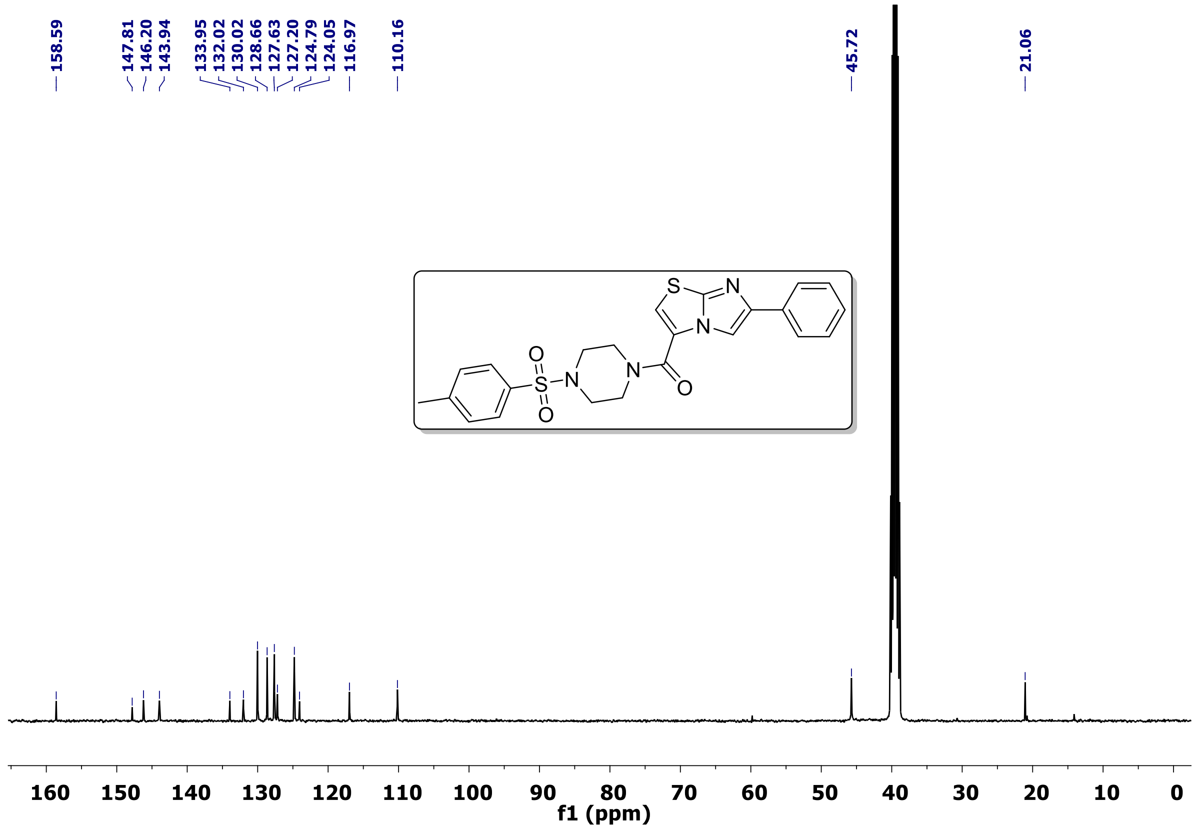

Copy of ^{1}H NMR and ^{13}C NMR spectra of **9ec**

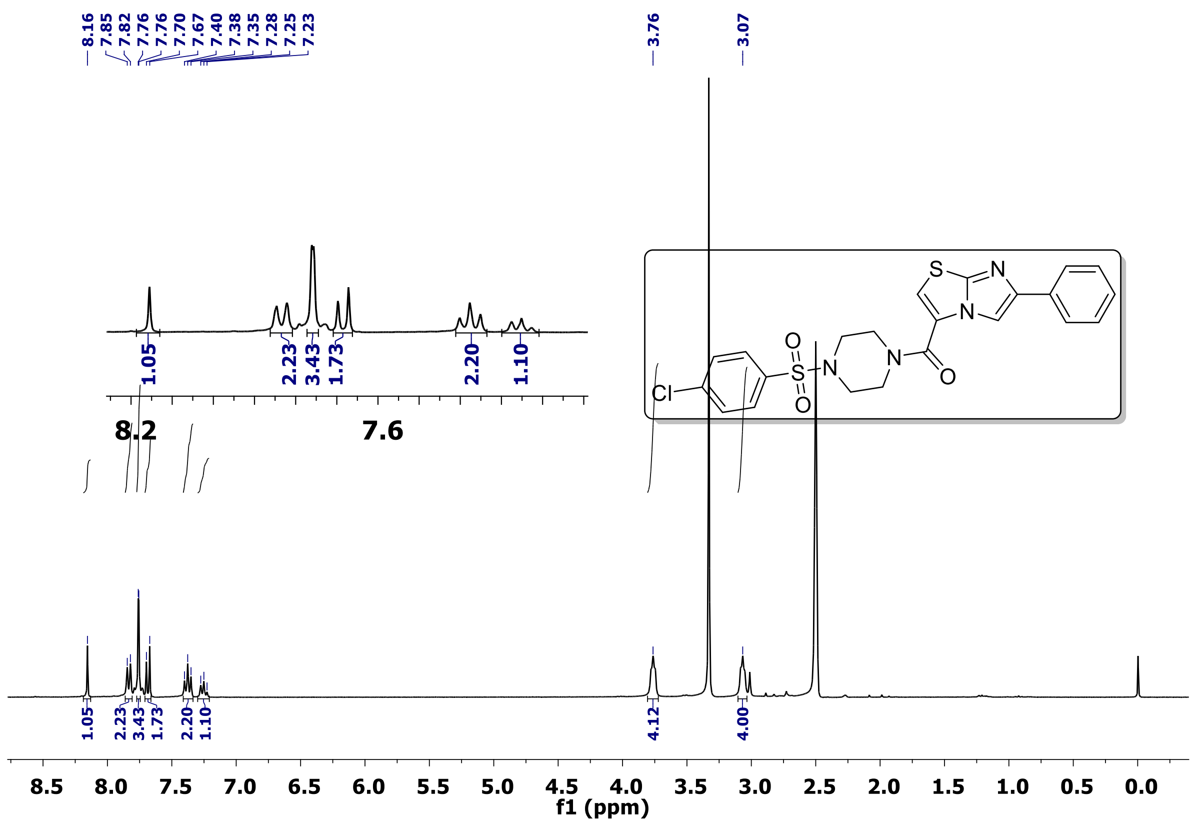

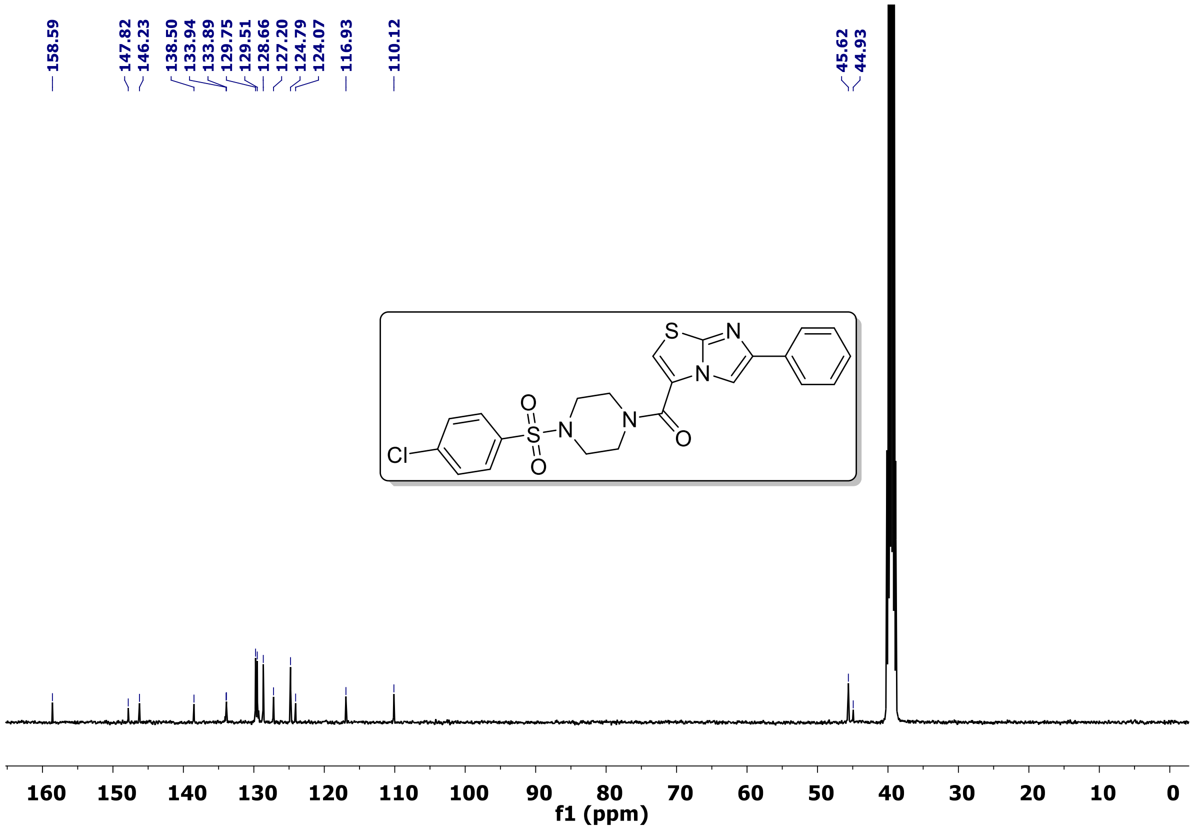

Copy of ^{1}H NMR and ^{13}C NMR spectra of **9ed**

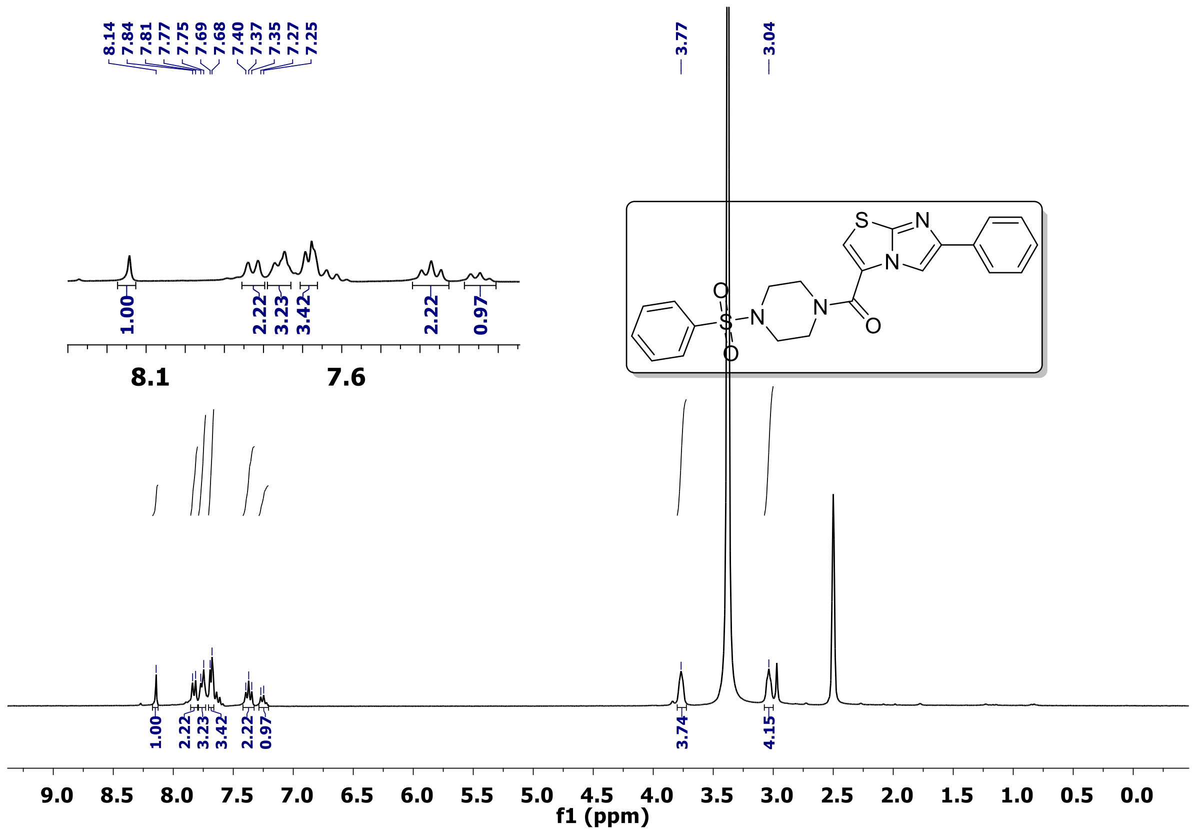

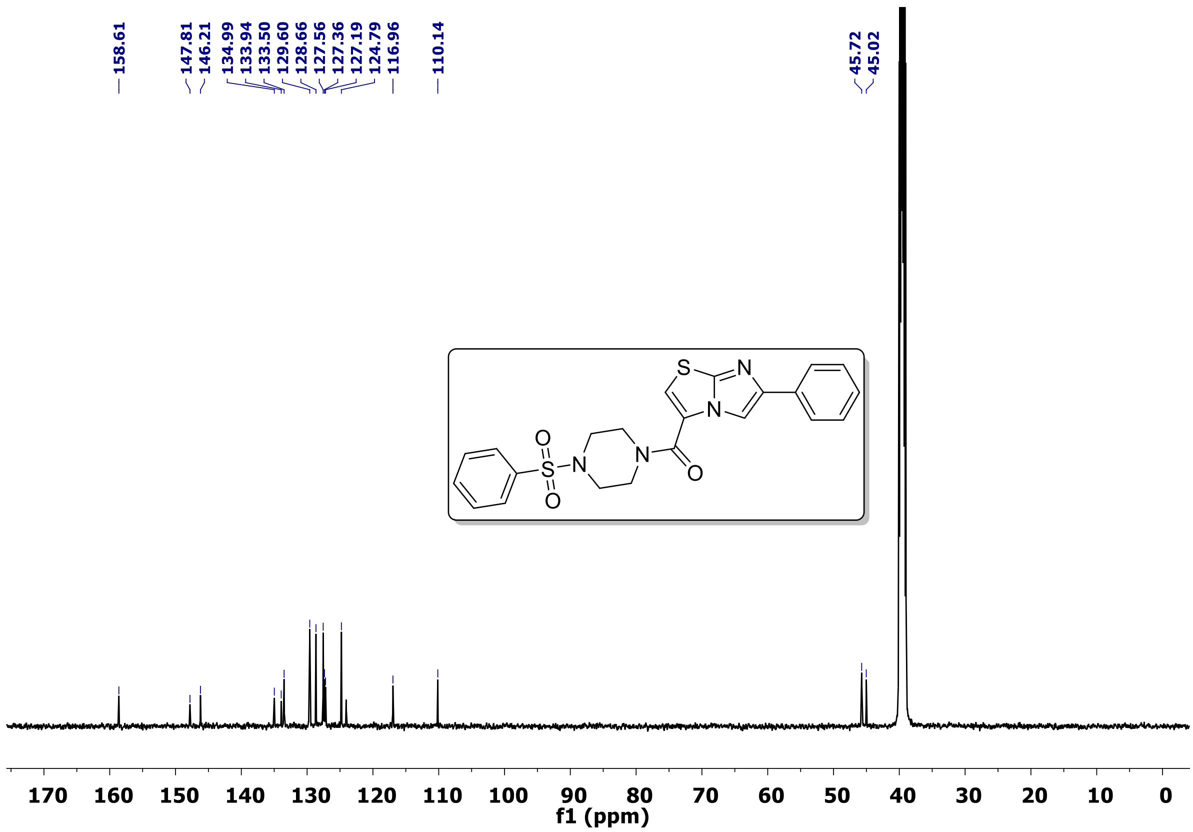

Copy of ^{1}H NMR and ^{13}C NMR spectra of **9ee**

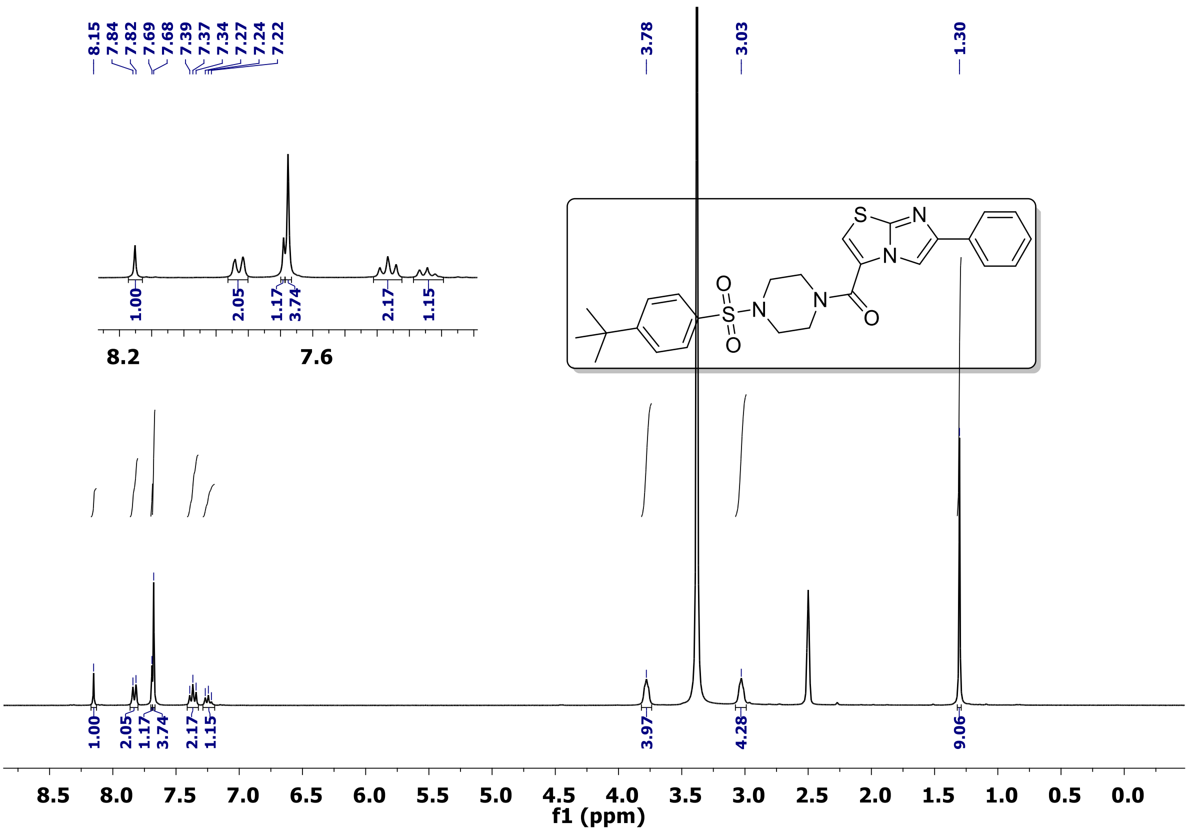

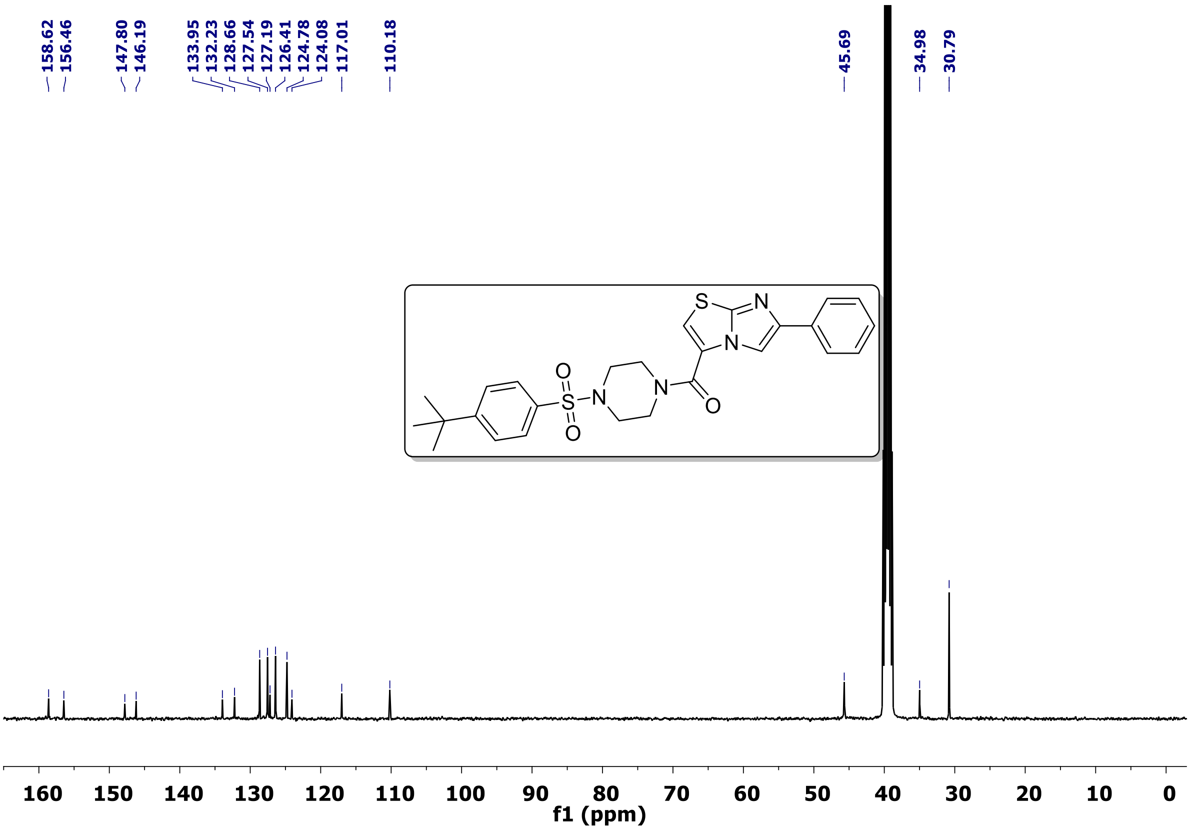

Supplement: Supplementary file 1 [file metabolites-10-00136-s001.pdf]
